# Gene Expression Response in Peripheral Blood Cells of Petroleum Workers Exposed to Sub-Ppm Benzene Levels

**DOI:** 10.3390/ijerph15112385

**Published:** 2018-10-27

**Authors:** Katarina M. Jørgensen, Ellen Færgestad Mosleth, Kristian Hovde Liland, Nancy B. Hopf, Rita Holdhus, Anne-Kristin Stavrum, Bjørn Tore Gjertsen, Jorunn Kirkeleit

**Affiliations:** 1 Department of Clinical Science, University of Bergen, P.O. Box 7804, N-5020 Bergen, Norway; katarina.joergensen@hi.no (K.M.J.); Rita.Holdhus@uib.no (R.H.); Anne-Kristin.Stavrum@uib.no (A.-K.S.); 2 Institute of Marine Research, P.O. Box 1870 Nordnes, N-5817 Bergen, Norway; 3 Nofima AS, Osloveien 1, N-1430 Ås, Norway; Ellen.Mosleth@nofima.no (E.F.M.); Kristian.Liland@nmbu.no (K.H.L.); 4 Faculty of Science and Technology, Norwegian University of Life Sciences, NO-1430 Ås, Norway; 5 Institute for Work and Health (IST), Universities of Lausanne and Geneva, CH-1066 Lausanne-Epalinges, Switzerland; Nancy.Hopf@hospvd.ch; 6 Department of Medical Genetics, Haukeland University Hospital, P.O. Box 1400, N-5021 Bergen, Norway; 7 Center for Cancer Biomarkers (CCBIO), Department of Clinical Science, Precision Oncology Research Group, University of Bergen, P.O. Box 7804, N-5020 Bergen, Norway; Bjorn.Gjertsen@uib.no; 8 Department of Global Public Health and Primary Care, University of Bergen, P.O. Box 7804, N-5020 Bergen, Norway

**Keywords:** benzene, gene expression, inflammation, immune response, leukaemia risk, petroleum industry, offshore

## Abstract

Altered gene expression in pathways relevant to leukaemogenesis, as well as reduced levels of circulating lymphocytes, have been reported in workers that were exposed to benzene concentrations below 1 ppm. In this study, we analysed whole blood global gene expression patterns in a worker cohort with altered levels of T cells and immunoglobulins IgM and IgA at three time points; pre-shift, post-shift (after three days), and post-recovery (12 hours later). Eight benzene exposed tank workers performing maintenance work in crude oil cargo tanks with a mean benzene exposure of 0.3 ppm (range 0.1–0.5 ppm) and five referents considered to be unexposed were examined by gene expression arrays. By using our data as independent validation, we reanalysed selected genes that were reported to be altered from previous studies of workers being exposed to sub-ppm benzene levels Four out of six genes previously proposed as marker genes in chronically exposed workers separated benzene exposed workers from unexposed referents (CLEC5, ACSL1, PRG2, IFNB1). Even better separation of benzene exposed workers and referents was observed for short-term exposure for genes in the Jak-STAT pathway, particularly elevated expression of IL6 and reduced expression of IL19.

## 1. Introduction

Benzene, a haematotoxic and leukaemogenic agent [1,2], is a natural component of crude oil and natural gas. Hence, workers handling the crude oil and production stream in the petroleum industry have a potential exposure to benzene. Haematotoxic effects from chronic benzene exposure include decreased white blood cell counts, circulating lymphocytes in particular [1], increased risk of acute myeloid leukaemia (AML) [2,3,4,5], and myelodysplastic syndrome [6], with increasing evidence also for multiple myeloma, chronic lymphocytic leukaemia, and non-Hodgkin lymphoma [5]. The haematotoxic effect and excess risk of AML have been reported among workers exposed to averaged benzene concentrations below 1 ppm [1,3,4], an exposure level previously considered not to cause any health effects. Benzene induced disruption of normal homeostasis in the bone marrow niche is suggested to precede cytogenetic injury and leukaemogenesis [6]. Gene expression studies of workers exposed to low levels of benzene (<1 ppm) with a dose-related decreased white blood cell counts, suggest that benzene also affects genes involved in AML and immune response pathways in a supra-linear manner [7], even at concentrations as low as 0.1 ppm benzene [8]. These observations were reported from a study of 125 Chinese subjects exposed to different levels of benzene, hereafter referred to as “the China benzene exposure study” [7,8,9]. An increased risk of haematopoietic malignancies and altered gene expression also at exposure below 1 ppm is compatible with a suggested non-linear metabolism of benzene, favouring production of a higher proportion of toxic metabolites in subjects exposed to benzene concentrations below 1 ppm than in heavily exposed workers [10]. Recently, six genes were selected from “the China benzene exposure study” as a minimal number of genes whose expression could predict low exposure to benzene (<1 ppm), including interferon beta 1 (IFNB1), acyl-CoA synthetase long chain family member 1 (ACSL1), nuclear factor kappa B subunit 1 (NFKB1), aquaporin 9 (AQP9), proteoglycan 2 (PRG2), and C-type lectin domain containing 5A (CLEC5A) [9].

In a longitudinal study of a small group of healthy petroleum workers that were exposed to benzene concentrations well below 1 ppm (geometric mean 0.15 ppm, range 0.01–0.62 ppm), we found a dose-dependent decline in the immune parameters IgA, IgM, and CD4^+^ T cell count after a period of three consecutive work shifts [11]. In the present study, we aimed to investigate whether these workers deviated from the unexposed referents also in the gene expression in whole blood samples. In order to limit the number of tests in our small data set, we utilized our data to perform an independent validation of the findings reported from “the China benzene exposure study”, by testing the six genes that were proposed by Schiffmann and co-workers (2018) to predict low levels of benzene exposure [9]. The Schiffman study also highlights the importance of immune and inflammatory genes in the response to benzene. In order to widen our search while keeping the numbers of genes analysed low, we selected a single pathway, the Jak-STAT signalling pathway, for a similar analysis. Jak-STAT is known to respond to interferons, cytokines, and hormones during inflammatory or immune responses and activates gene expression directing cellular processes such as cell death, proliferation and differentiation [12]. This pathway is known to play an important role in both preleukemic and leukemic cells [13,14], and was previously reported by McHale and co-workers (2011) to be affected by low dose benzene exposure [7]. We tested these genes in data with and without fold change to investigate differences in gene expression between referents and exposed petroleum workers both as results of exposure prior to the experimental period of the present study and as a result of exposure resulting from a three-day period of shift work with observed exposure of low doses of benzene.

## 2. Materials and Methods

We conducted the study with the approval of the Regional Committee for Medical Research Ethics of Western Norway (REC West no. 102.04) and the Norwegian Social Science Data Services (project no. 11187). The Directorate for Health and Social Affairs gave permission to establish a biobank. Participation was voluntary, and informed written consent was obtained from all participants. All of the subjects were informed about their own test results on exposure and haematological parameters both in personal letters, and if follow-up was recommended, the participants were also informed by oral communication. 

**Figure 1:**
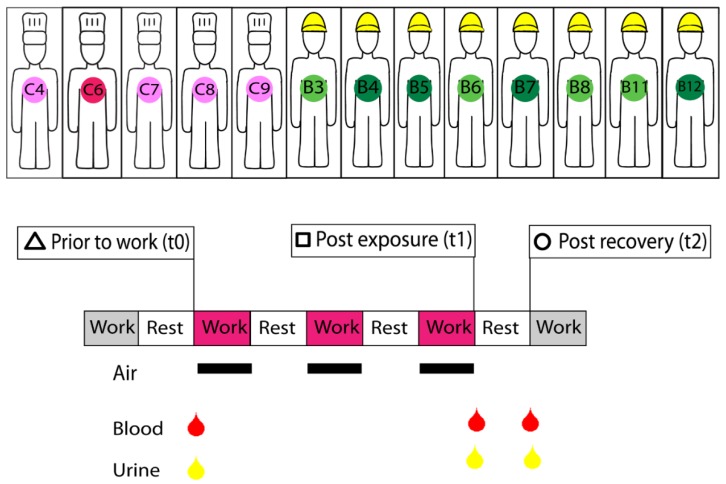
Study design. Biological samples were collected from five unexposed offshore caterers (left, pink) and eight exposed (right, green) tank workers. Lighter shades represent workers in the age balanced dataset while darker shades denote the individuals not included. Samples were taken before work shift (time 0, t_0_), after three consecutive work shifts (time 1, t_1_) and again after recovery of 12 h (time 2, t_2_). Benzene concentration in tank workers’ breathing zone air was measured by passive dosimeter badges during all three work shifts. Benzene concentration was measured in blood and urine at all three sampling time points. RNA was extracted from blood taken at all three time points. Other parameters measured in blood include toluene, differential cell counts, immunoglobulins, and complement components (see Supplementary Table S2) and in urine toluene, t,t-muconic acid, and 1-hydroxypyrene. Information on each tank workers’ total time spent in the tank was also noted.

The study population consisted of nine men that were exposed to benzene during maintenance work in crude oil cargo tanks on a production vessel on the Norwegian continental shelf and six referents recruited from the catering section on the same vessel, who were considered to be unexposed to benzene (Figure 1). The tank work included tank inspection, scaffold construction, and welding to mend leaks in crude oil cargo tanks (volume 5000–7800 m^3^). Before maintenance work started, the tanks were cleaned with hot crude oil and sea water and purged with inert gases and fresh air. The empty storage vessels ready for inspection and maintenance had sludge lining the inside of the tank. Hence, the tanks were continuously ventilated with forced air. Use of protective suits (Tyvek^®^) and half-face air-purifying respirators with a combination of particle filter and organic vapor cartridge were mandatory during cleaning and inspection, and the chemical protective suits were worn over the cotton coveralls and were discarded twice during each shift. During scaffold building and welding, the use of respirators varied, but was not systematically recorded. Catering (reference population) included work in the canteen and housekeeping (cleaning of the living quarter and some laundry). The referents spent the whole work period inside the living quarters, and although one of them was exposed to cooking fumes and one was potentially exposed to crude oil residues during cleaning of dirty suits, none of the referents were in the processing area where crude oil or benzene-containing produced water was present. During the study period, all subjects lived on board the production vessel and were exposed to the same environment in the living areas and had similar work schedules and diet. Smoking was prohibited in the processing area, but the living quarter had a limited number of designated smoking rooms. Since alcohol was banned on the installation, no participant consumed alcohol during the study period.

The sampling period was three consecutive 12-h work shifts (Figure 1). The first sample was collected in the morning (0700–0900) before the workers entered the tank (prior to the work shifts, t_0_) and it was considered a baseline measurement. The second sample was collected at the end of the work shift (1800–2100) on the third day of tank work (post exposure, t_1_), and a third sample was collected in the morning (0700–0900) the following day (post recovery, t_2_). Blood and urine samples were obtained from the referents on the same days following the same time protocol. The participants completed a self-administrated questionnaire including questions on age, sex, medication use, and whether they were current smokers during the study period (Table 1 and Supplementary Table S1a,b). Time spent in tank was registered by the operator in the central control room. During the days prior to the sampling period workers performed preparatory work with only minor exposure to benzene [15]. The data are first analysed with fold change by taking the observation at the end of the work shift (t_1_) and the following morning (t_2_) as a ratio of the time point prior to expose (t_0_), which gave two time-points of fold change data for each person. Secondly, the data were analysed without fold change at each time point.

The original study population [11,15] comprised 12 tank workers that were exposed to benzene (denoted B) and nine referents (denoted C), but, in the present study, we only included the workers providing three consecutively collected blood and urine samples. In addition, two individuals were excluded due to medical reasons (C5 and B10); C5 was diagnosed with cancer approximately one year after the data collection and B10 left the tank work due to back pain on the first day of the study period being administered diclofenac. After excluding C5 and B10, the final study population comprised eight exposed workers and five referents (Figure 1).

**Figure 2:**
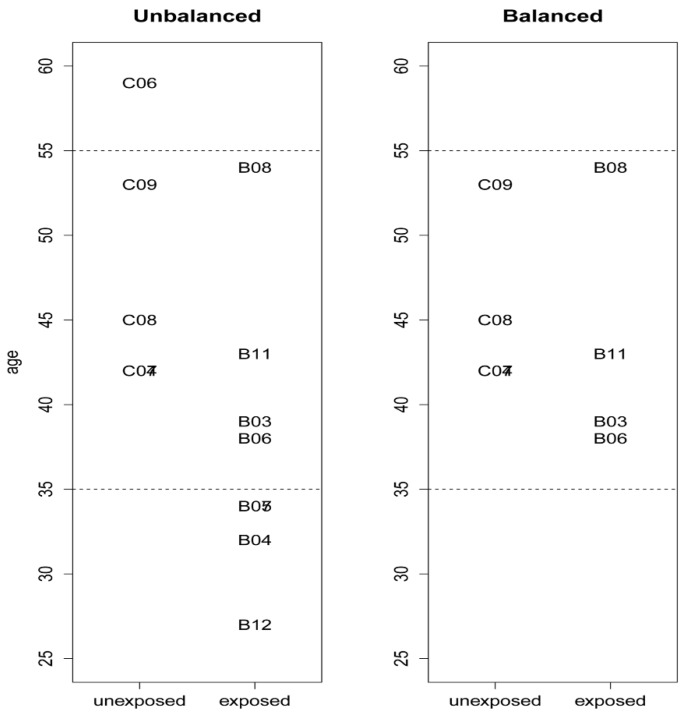
Distribution of age for the complete and age-balanced dataset. Benzene exposed (B), and referents (control group (C)). Two of the referents (C04 and C07) and two exposed workers (B05 and B07) are overlapping in the figure as they had the same age.

Average age was found to be significantly different between the two groups. An age balanced dataset was therefore generated by excluding workers at age < 35 and >55 (see Figure 1 and Figure 2). The age-balanced data set was used to select genes affected by benzene exposure without a confounding influence of age using Elastic Net analysis. The balanced data set included the exposed workers: B3, B6, B8, and B11; and referents: C4, C7, C8, and C9. Both the balanced and unbalanced data were, however, utilised in explorative Principal Component Analysis (PCA), and plots of selected genes.

The workers’ environmental and biological concentrations of relevant petroleum-derived hydrocarbons were sampled according to the protocol given in Figure 1. The strategy for exposure assessment and methods of analysis of markers of exposure to benzene, toluene, ethylbenzene, xylene (BTEX) and the polycyclic aromatic hydrocarbon (PAH) pyrene have been described elsewhere [15,16]. In short, the tank workers’ personal exposure to BTEX was measured during three consecutive shifts using organic vapour passive dosimeter badges (3M 3500^TM^) attached in the worker’s breathing zone (the worker’s lapel). Personal exposure to BTEX was not measured for the reference group as they were assumed not to be exposed to benzene above the background concentration in the indoor environment. The dosimeter badges were desorbed with CS_2_, and were analysed quantitatively and qualitatively by gas chromatography with mass spectrometry (NIOSH, 2003). The limits of detection were 0.001 ppm for benzene and 0.01 ppm for toluene, ethylbenzene and xylene. Blood samples for determining benzene and toluene were collected by venipuncture into Venoject II^®^ tubes (hard plastic) with heparin, and the urine samples for the determination of benzene and toluene, t,t-muconic acid (t,t-MA), and 1-hydroxypyrene (1-HP, a pyrene metabolite) were collected in glass bottles (Pyrex^®^) with polypropylene stoppers. The samples were stored at 4 °C until they were transported to the Biomonitoring Laboratory at the Finnish Institute of Occupational Health in Helsinki, Finland (benzene and toluene in blood and urine and t,t-MA in urine) and frozen at −20 °C to the University of Cincinnati, Cincinnati, OH. USA (urinary 1-HP), for analysis. The benzene and toluene concentrations in blood were analysed by a head-space sampler (Perkin Elmer Headspace sampler HS40) and a gas chromatograph (Perkin Elmer Autosystem Gas Chromatograph) using photoionization detection according to the method described by Pekari et al. [17]. Samples with benzene levels at or below 5 nmol/L were analysed by multi-head space extraction [18]. Urinary benzene and toluene concentrations were analysed using a solid phase micro-extraction–gas chromatograph–iontrap method (SPME-GC-Iontrap-method), while t,t-MA was extracted from urine by solid phase extraction in an ion exchange column, and was analysed by a liquid chromatography with ultraviolet detection method [16]. Urinary 1-HP was analysed in frozen urine samples by high performance liquid chromatography (HPLC) with fluorescence detection after enzymatic hydrolysis [19]. The limits of quantification were 1 nmol/L for benzene and toluene in blood and urine, 0.5 µmol/L for t,t-MA and 12 ng/L for 1-HP. All values below the limit of quantification and benzene exposure in referents were replaced by values that were equal to the limit of quantification divided by 2 [20].

Blood samples for collection of RNA were drawn by venipuncture into PAXgene Blood RNA tubes^®^ and total RNA from the human whole blood were extracted by using the PAXgene Blood RNA Kit from PreAnalytiX/Qiagen. Amount (9.7–97 µg) and quality of the extracted RNA was verified by the NanoDrop^®^ ND-1000 spectrophotometer (NanoDrop Technologies, Wilmington, DE. USA) and the Agilent 2100 Bioanalyzer (Agilent Technologies, Santa Clara, CA. USA). Paxgene tubes were stored at −20 °C prior to extraction of RNA, but had an acceptable RNA quality with Integrity Number (RIN)-values of RNA ≤ 7.2.

All microarray experiments were performed using the Illumina BeadStation 500GX, which is based upon fluorescence detection of biotin labelled complimentary RNA (cRNA). 500 ng of total RNA from each sample was reversely transcribed, amplified, and Biotin-16-UTP –labelled, using the Illumina TotalPrep RNA Amplification Kit (Applied Biosystems/Ambion, Waltham, MA. USA).

750 ng of biotin labelled cRNA was hybridized to the HumanRef-8 v3 Illumina Sentrix BeadChip according to manufacturer’s instructions. The HumanRef-8 v3 BeadChip targets approximately 24,500 well annotated RefSeq transcripts and covers 18,631 unique curated genes. The samples were labelled and hybridised in two batches, but care was taken to ensure that all the samples from the same individual were handled in the same batch in each step. The samples were randomly distributed on the slides, 8 samples on each slide.

The data from the Illumina BeadStudio v.3.3.7 software was quality controlled using R version 2.8.1 [21]. Prior to importing data to BeadStudio the data was summarised from bead level to probe level by the BeadArray reader. Before being compiled into an expression profile data matrix, all arrays were quantile normalised to be comparable. First, Bead Array scanning (Illumina) was used to verify successful scanning. Bead Studio was then used to carry out the built-in quality checks against internal controls in the array. Next, J-Express Pro v 2.7 [22] was used to check for outliers and batch effects, including use of density plot, box plot, correspondence analysis, and hierarchical clustering with a Pearson distance matrix.

The gene expression data was log transformed, and changes in the gene expression data from prior exposure to after exposure were calculated as fold change (FC) data post-shift (time 1, t_1_) and pre-next shift (time 2, t_2_) over pre-shift (time 0, t_0_). As the fold change ratio was log transformed for normalisation fold change becomes a difference as: FCt_1_ = log2(t_1_/t_0_) = log2(t_1_) − log2(t_0_), and likewise for FCt_2_. The microarray data are available in ArrayExpress, access number E-MTAB-5331, where they have been described according to MIAME principles [23].

Illumina ID and gene name conversions for this study (IlluminaHumanRef8v3) and “the China benzene exposure study” [7,8,9] (IlluminaHumanRef8v2) were performed using reference lists for the arrays posted in GEO (GPL6883-11606 and GPL6104-11576) and using gConvert, a part of the gProfiler tool [24] in R (version 3.5.0).

**Figure 3:**
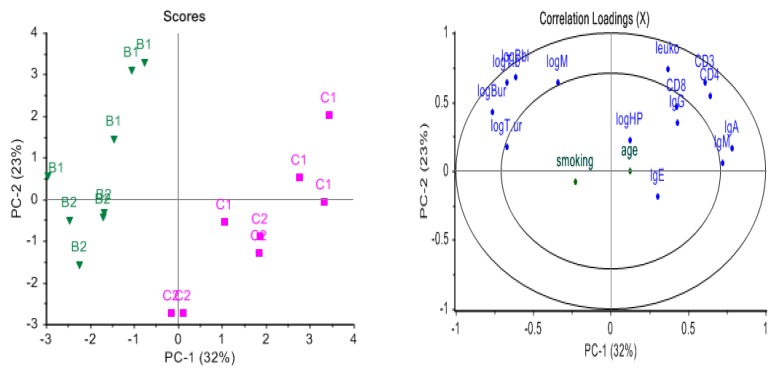
Explorative multivariate analysis, Principal Component Analysis (PCA) of the fold change of descriptive data between time 1 and 2 for the age balanced dataset, where all features were standardized to unit variance. (**a**) score plot of the samples and (**b**) corresponding loading plot. Benzene exposed subjects are shown to the left in green (B) and referents to the right in pink (C), where 1 = time 1 (post-shift), 2 = time 2 (pre-next shift). The variables that clustered on top of each other towards to the left in the figure were more highly expressed in benzene exposed workers, comprising benzene and toluene in blood and urine, as well as 1-hydroxypyrene (HP) and t,t-muconic acid (muconic).

Characteristics of the study population (sex, age, number of smokers) and measures of exposure at post shift (t_1_) (BTEX in air, benzene and toluene in blood and urine, urinary t,t-MA and urinary 1-HP) were presented and used without any transformations (Table 1 and Supplementary Table S2). The observed variation in the descriptive analysis was analysed exploratively by principal component analysis (PCA). All variables were standardized to unit variance to allow all to have the same influence on the model. Sex, age, and smoking were down-weighted, so that these variables, although shown in the correlation plot, did not affect the model (Figure 3).

PCA was used to visualize the relationship between chemical exposure and immunoglobulin levels and leukocyte counts. PCA identifies the relationships that explain as much of the variance in the data as possible and separates these into orthogonal principal components [25]. The analysis was performed in Unscrambler (CAMO Software AS (version: 10.3.31813.89), Oslo, Norway. https://www.camo.com/unscrambler).

Next, supervised multivariate analysis was performed using gene expression data as input and benzene exposure vs. referents as response (as a categorical variable). The first analysis was performed on the six genes Schiffmann and coworkers [9] selected from the “the China benzene exposure study” as the minimal number of genes for prediction of benzene exposure below 1 ppm. We also analysed all 30 of the genes tested by Schiffman and coworkers [9]. Finally, a separate analysis was performed on 162 genes included in the Jak-STAT pathway, as defined by KEGG (hsa04630) using Enrichr [26,27], a pathway reported by McHale and co-workers [7] to be affected by low benzene exposure.

Elastic Net (R package glmnet version 2.0-16) were used for the analysis, and genes selected in each model were recorded [28,29]. Elastic Net uses a general linear model with penalty terms for feature selection. The method is a combination of ridge regression (RR) and least absolute shrinkage and selection operator (LASSO). Both methods add a small constant penalty term, *λ*, into the algorithm to make the estimated regression coefficients more stable. Adding the penalty term may reduce overfitting and improve the prediction error. RR does not perform variable selection since none of the input terms are downscaled to zero, whereas LASSO performs both variable selection and regularization in order to enhance the prediction accuracy and improve the interpretability of the statistical model. Elastic Net employs a predefined tuning parameter, *α*, to define the balance between the RR and LASSO methods. Elastic Nets produce sparse models aiming for optimal prediction [28]. Elastic Net analysis was performed using tuning parameter *α* = 0.5, and *λ* was set to “*λ* minimum”. The model was performed within each time point and validated using full cross validation where one patient at the time is omitted from the calibration and used for validation. The analyses were run with fold change on each of the two time points; immediately after three consecutive work shifts (time 1) and again after recovery of 12 h (time 2) over time point 0, prior to benzene exposure, and without fold change at each time point.

PCA [25] was also used to illustrate the combined ability of the genes selected by Elastic Net to separate the two exposure groups (performed by singular value decomposition coded longhand in R 3.5.1.Standardization was used). All data at each time point was included in the model, also patients excluded in the age balanced data set.

Technical verification of individual genes (e.g., polymerase chain reaction (PCR)) was not included since the Illumina array used in the present study has previously been shown to be highly reliable [30]. PCR is a technical validation of array quality and probe reliability. We here perform a genuine biological verification by using genes previously found to predict benzene exposure and one pathway previously reported to be affected by low dose of benzene [7,9].

## 3. Results

### 3.1. Study Population and Exposure

Characteristics of the study population and descriptive statistics of environmental hydrocarbon exposure on the third day of study and concentrations in blood and urine post shift are given in Table 1. The raw data is appended as supplementary information (Supplementary Table S2). Tank workers’ arithmetic mean exposure to benzene was 0.25 ppm (range 0.08–0.50 ppm) in the balanced dataset, somewhat higher than in the unbalanced data set. In the balanced data set, all four tank workers were former smokers, while the referents comprised one current smoker, one former smoker and two never-smokers.

PCA on the descriptor variables (leukocyte cell counts, immunoglobulins, chemical exposure) on data with fold change performed on the age balanced data set is displayed in Figure 3. The concentration of benzene and toluene in blood and urine were all located towards the left in the loading plot, at the same side as benzene exposed workers. The immunoglobulins were located towards the opposite side, being negatively associated with benzene exposure. Age, and smoking status were down-weighted in the analysis so that it did not affect the analysis. Figure 3 illustrates that these variables, located in the centre of the plot, were not related to the differences between the groups in the age-balanced data set.

### 3.2. Selecting Marker Genes Predictive of Exposure Group at Current Exposure

**Figure 4:**
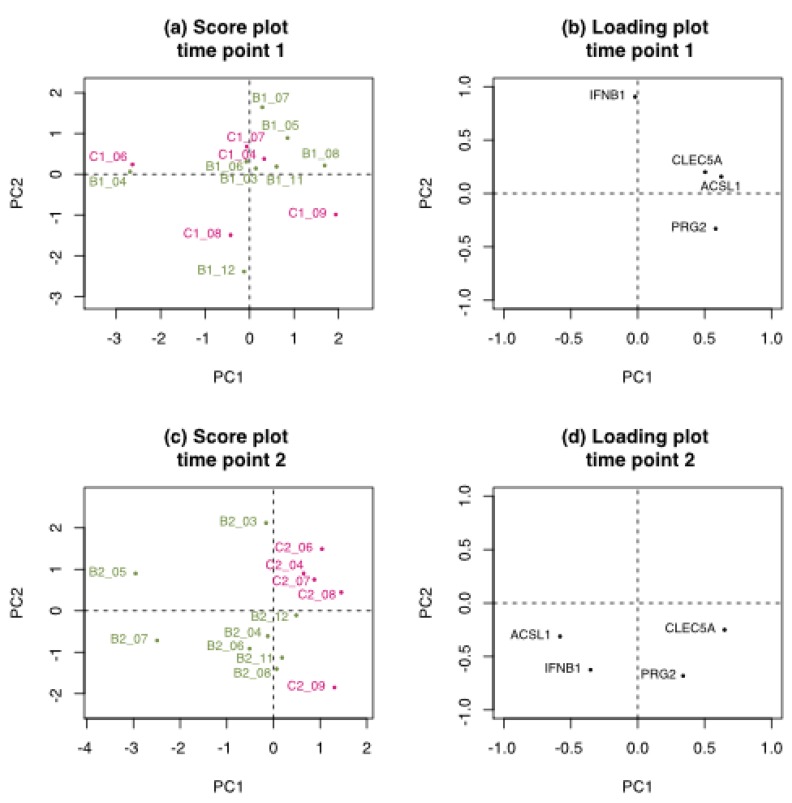
PCA performed using four genes selected by Elastic Net marker using the six genes proposed from “the China benzene exposure study” as markers of Benzene exposure. Each time point was run separately as fold change over time 0, prior exposure. Time point 1 (**a**,**b**) immediately after 3 days of benzene exposure (post-shift), and time point 2 (**c,d**) one day after recovery(pre-next shift). Scores (**a**,**c**) of exposed workers (in green), referents (in pink), and corresponding loadings of the genes (**b**,**d**).

Elastic Net analysis selected four of the six genes identified as markers predicting low benzene exposure (below 1 ppm) from “the China benzene exposure study” [9] on data with fold change at time point 2 (pre-next shift), but not at time point 1 (post-shift) (Figure 4 and Supplementary Figure S1, the former showing the four selected genes and the latter all six). The selected genes include PRG2, IFNB1, ACSL1 and CLEC5A. Genes not selected in the present study were AQP9 and NFKB1 (see Supplementary Table S3 for Gene ID list). The PCA model of all six genes shows that the exposure groups did not separate at time point 1 (Figure 4a (four selected genes) and Supplementary Figure S2a (all six genes)), explaining the lack of model in the Elastic Net analysis at time point 1. At time point 2 (Figure 4c and Figure S2c), the referents (pink) and exposed workers (green) were more separated based on the expression of the selected gene, but the model with just the four selected genes was clearly the better of the two. The genes PRG2 and CLEC5A (and AQP9) tended to be more highly expressed in referents at time 2, whereas ACSL1 and IFNB1 (and NFKB1) were more highly expressed in exposed workers (Figure 4d (four selected genes) and Supplementary Figure S2d (all six genes)). Individual plots of the expression of the six genes in all workers can be found in Supplementary Figure S3 showing that the gene expression of CLEC5A was the gene that most strongly separated Benzene exposed vs. referents when considering one gene at the time among these six genes.

For comparison, we also ran all 30 genes selected for testing by Schiffman [9], and this resulted in twelve selected genes at time 2 (ACSL1, CD44, CLEC5A, GPR132, IFNB1, IL1A, IL6, IL1RN, MPL, PLAUR (ILMN_1691508 and ILMN_2374340), PTX3, ZNF703), and none at time 1 (see Supplementary Table S4 for gene IDs, Supplementary Figure S4 Elastic Net and PCA models). This model gives clear separation of the referents and exposed workers at time point 2. Note that three of the original six marker genes were selected (ACSL1, CLEC5A, IFNB1) when Elastic Net was given a wider choice.

**Figure 5:**
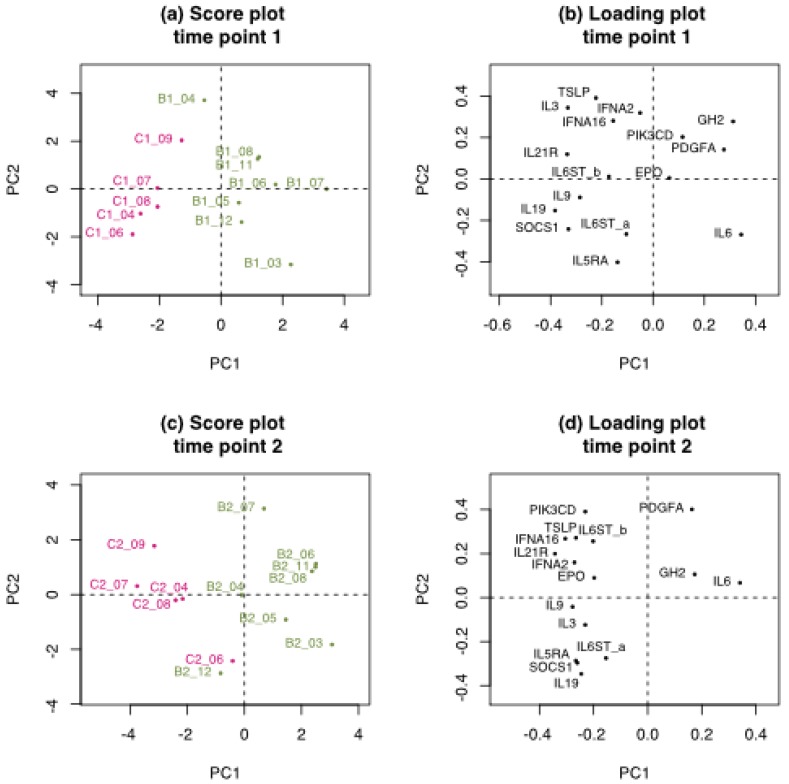
Principal Component Analysis (PCA) performed using sixteen genes selected by Elastic Net from the Jak-STAT pathway (Hsa04630). Each time point was run separately as fold change over time 0, prior exposure. Time point 1 (**a**,**b**) immediately after three days of benzene exposure (post-shift), and time point 2 (**c**,**d**) one day after recovery(pre-next shift). Scores (**a,c**) of exposed workers (in green), referents (in pink), and corresponding loadings of the genes (**b**,**d**).

The Elastic Net analysis performed with genes belonging to the Jak-STAT pathway on the age-balanced data set selected 16 out of 162 genes reported to predict exposure to benzene [9] (Supplementary Figure S5). As seen in the analysis of the six proposed marker genes from the study of Schiffmann and coworkers [9], the Jak-STAT genes were also selected at time point 2 (pre-next shift), but not at time point 1 (post-shift). Although, for the Jak-STAT genes, PCA analysis of the selected genes showed that the exposure groups separated fairly well, even at time point 1 (Figure 5). At time point 2, the workers separated somewhat more clearly into exposed workers and unexposed referents (Figure 5c), and the genes also separated into two distinct groups being relatively highly expressed in either referents (IL9, SOCS1, EPO, TSLP, IFNA16, IL19, IL3, IL6ST_a (ILMN_1746604), IL5RA, PIK3CD, IL6ST_b (ILMN_1797861), IL21R, IFNA2) or exposed workers (IL6, GH2, PDGFA) (see Supplementary Table S5 for full Gene IDs). Most of the selected genes are effectors of the Jak-STAT pathway; interferons, interleukins, and their interactors or hormones. Furthermore, plots of the expression of each gene selected in the Jak-STAT pathway at time point 2 illustrate that several genes individually show a distinct separation of the expression levels in referents (pink C) and exposed workers (green B) (Supplementary Figure S6). Among the Schiffman genes, ACSL1 and CLEC5 separated the groups well on their own on the data with fold change, whereas IL6 and IL19 were the best pair among the Jak-STAT genes (Supplementary Figure S7, page 1).

### 3.3. Considering Gene Expression Differences in Worker Groups Prior to Exposure

To provide a more in-depth understanding of the responses, we repeated our analyses on genes without fold change at each time point separately (time 0, time 1, and time 2). As before, Elastic Net analyses were performed using exposure group as the response (referents vs. exposed workers), followed by PCA and gene plotting. No models were selected at any time point when just the six China marker genes were used (not shown). When all 30 genes were used, eight were selected at time 0 (ACSL1, IL6, IL1RN, IFNB1, CEBPA, CD44 (ILMN_1778625 and ILMN_2348788), ZNF703, TTC9B), and two at time 1 (ACSL1, IL1RN). (see Supplementary Table S6 for gene IDs, Supplementary Figure S8 Elastic Net and PCA models and individual gene plots). Four of the genes (IFNB1, ACSL1, IL6 and IL1RN) were selected both at time 0 in data without fold change (Supplementary Figure S8) and at time 2 in data with fold change (Supplementary Figure S4). Similarly, the Jak-STAT pathway analysed without fold change yielded a model of 10 genes at time 0 (IL3, IL6, IL19, IL24, IFNA13, CSH1/CSH2, SOCS1, JAK1, PIAS2, SOS1), and two at time 2 (EPOR, IL15). (Supplementary Table S7 for gene IDs, Supplementary Figure S9 Elastic Net and PCA models and individual gene plots). Here, genes also selected at time 2 in data with fold change (Figure 5) were IL3, IL6, and IL19. Our gene pairs CLEC5/ACSL1, IL6/IL19 and a new choice based on the above finding: IL6/SOCS1 did separate the workers distinctly at both time 0 without fold change and at time 2 with fold change (Supplementary Figure S7).

**Figure 6:**
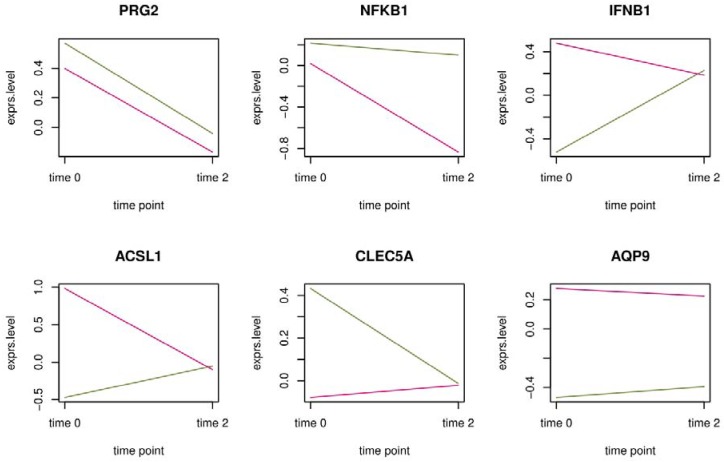
Time series plots of gene expression without fold change for the six marker genes proposed by the China benzene exposure study. Pink: Average gene expression in referents. Green: Average gene expression in exposed workers.

To inspect the expression of individual genes without fold change selected by Elastic Net, time series plots of average gene expression were chosen. The time trends in the six marker genes that were proposed by Schiffman and coworkers [9] (Figure 6) show that many genes selected had opposite trends in referents (pink) and exposed workers (green). A wider selection of potential marker genes (20 in total) from this study were included in Supplementary Figure S10. Elastic Net chooses genes based on the difference in average gene expression between referents and exposed workers. Confidence intervals based on this difference for the same selection of marker genes can be found in Supplementary Figure S11 for data without fold change at time 0 and for data with fold change at time point 2.

## 4. Discussion

Benzene exposed workers, which were previously reported to have a dose-dependent decline in several immune parameters, including CD4^+^ T cell count [11], deviated from unexposed referents also in the gene expression pattern in peripheral blood. The results agree with observations in previous studies of gene expression in workers with comparable low benzene exposure (<1 ppm) [7,8,9]. We found that four out of six genes that were previously suggested to predict benzene exposure in this range [9] were selected by Elastic Net as a minimal predictive model also in the present study, and they separated benzene exposed workers from referents by the two first PCs in the explorative PCA analysis. Furthermore, we found that genes that are associated with the Jak-STAT pathway, also detected in a previous study [7], may additionally predict low benzene exposures. Exposure to benzene at mean concentrations up to 0.02 ppm, being approximately one tenth of the mean concentration reported in the present study, has also been reported to be associated with altered DNA methylation patterns [31,32]. Hence, together with observations in other studies on workers exposed to low concentrations of benzene, our finding supports that peripheral blood cells respond on a molecular level to benzene concentrations at sub-ppm levels. By using our data as a different data set, these results constitute independent support for the benzene-induced alteration of disease-relevant genes and pathways previously reported among benzene-exposed Chinese workers [7,8,9].

Unlike a univariate approach, which aims to find as many differentially expressed genes as possible, the purpose of Elastic Net is to identify genes that together would give optimal prediction of exposure group (unexposed vs. exposed). The finding that four out of the six proposed marker genes were selected by Elastic Net suggests that, in our data set, all four are required for prediction. C-type lectin domain family 5A (CLEC5A) was the best individual predictor in this study among the previously proposed marker genes, but also worked well together with acyl-CoA synthetase long chain family member 1 (ACSL1). CLEC5A is a myeloid differentiation gene known to participate in inflammatory responses [33]. Although Elastic Net selects a greater number of Jak-STAT genes (16), the clarity of the separation of the groups as well as the ability of several of the individual genes to separate groups, suggests that these are a better choice in our dataset at the pre-next shift time point (t_2_). Interleukins IL-6 and IL-19, in particular, showed a strong ability to separate the workers (Supplementary Figure S6). IL-6 was a candidate gene in the Schiffman study [9], however it was not selected as a final proposed predictor gene. We suggest to include IL-6and IL-19 and perhaps other Jak-STAT associated genes in future studies aiming to identify markers of short-term benzene exposure.

Our data included three time points of measurements, one prior to (pre-shift) and two time points after the start of benzene exposure (post-shift and pre-next shift). Most of the genes change their expression over time, suggesting that longitudinal data collection with repeated measures can detect effects that add more information than only one time point. In the “The China benzene exposure” study [7,8,9] the biological samples were collected at the beginning of the work shift (pre-shift) [34], hence being comparable to our pre-shift and pre-next shift samples (time points 0 and 2). The occupational history of our exposed workers was not known in detail and they may have had different levels of past exposures to petrochemicals in the previous weeks and months, which may in fact affect their baseline gene expression, since it is known that benzene exposure may change gene methylation patterns [31,32]. In contrast, the referents are caterers and probably do not have any history of such past occupational exposure. Hence, our approach aimed mainly at identifying responses due to current exposure. The China benzene study is more geared towards selecting genes that reflect chronic exposure, i.e., finding differences in gene expression, regardless of baseline expression [9]. This is an equally important goal that is suitable for a different target group. Secondly, we note that Elastic Net picks out prediction models mainly at time point 2 in the data with fold change, suggesting that the gene expression response may take time to develop. This is consistent with findings in gene expression time series experiments in laboratory settings [35]. Interestingly, when we analysed the genes without fold change, Elastic Net found models mainly at time 0. Gene expression patterns were thus different already before exposure in the referents and exposed workers. Some genes (IFNB1, ACSL1, CD44, IL6, IL1RN, ZNF703, IL3, SOCS1 and IL19) were selected both at time 0 in gene data without fold change as well as at time 2 in data with fold change. There are many potential reasons for the differences between the studies, however the Chinese workers are more likely to have been routinely exposed to <1 ppm benzene in the workplace over a prolonged period of time. Our result reflected also short-term exposure, and it is interesting to note that even this short exposure period results in responses that can separate the exposed workers from the unexposed. Marker genes should ideally be valid over a range of naturally occurring conditions in order to have a broad-based use value. Hence, the potential effect of both sampling time and exposure history on the optimal choice of marker genes are worthy of further exploration.

Although this small and exploratory study was not designed to explain a possible correlation between low level benzene exposure and reported excess risk of hematopoietic malignancies in this group of workers [36,37,38], our and others’ observations [7,8] may point to possible mechanisms involved. The selection of many effectors of the Jak-STAT pathway as predictive of exposure group at time 2 analysed with fold change is particularly intriguing, given its known role in leukaemogenesis [13]. The majority of the selected genes are downregulated in exposed workers when compared to referents as considered by fold change values reflecting short-term exposure, while interleukin 6 (IL-6), platelet-derived growth factor subunit A (PDGFA), and growth hormone 2 (GH2) were upregulated in exposed workers. IL-3, IL-6, and erythropoietin (EPO) all have known roles in haematopoiesis, the pathway that becomes dysregulated in leukaemogenesis [13]. Suppressor of cytokine signalling 1 (SOCS1) is a feedback regulator, and regulation of its expression is thought to play a role in the dysregulation of Jak-STAT signalling [39]. However, its role in myelodysplastic syndrome and acute myeloid leukaemia and their development is still not fully understood [40]. 

Analysis of age at exposure in a cohort of workers producing rubberized cloth, the so-called Pliofilm cohort, suggested that the effect of benzene on leukaemia mortality is of greater magnitude for workers exposed at older ages (>45) than for those exposed at younger ages [41]. However, the genes that were selected as differently affected in exposed workers compared to referents over the specified time course were not influenced by age as the data used in the prediction model was balanced by age, and our PCA analysis supports this (Figure 3).

Cigarette smoke contains benzene; hence, smoking is a potential confounder for occupational exposure to benzene [42]. Most of the study subjects were former smokers, and the only current smoker was in the control group. Analysis showed that smoking did not separate referents from exposed workers in our data (Figure 3).

Benzene and toluene are simultaneously present in crude tank; hence, co-exposure occurred in the present cohort. Although the mean toluene exposure was as low as 3% of the Norwegian occupational exposure limit (25 ppm), the internal concentration of unmetabolized toluene was significantly elevated in exposed workers as compared to referents. A few studies reporting on benzene’s haematotoxic effects in workers also report co-exposure with toluene [1,43]. While one concluded that toluene exposure had little effect on the reported haematotoxic effects [1], another reported that toluene was seen to significantly confound the findings for many of the cell populations, including lymphocytes and neutrophils [43]. Further, concomitant toluene exposure has also been reported to moderate benzene’s metabolism and haematotoxic effect [44], and existing evidence suggests that the direction of the modifying effect is dose-dependent. The concentrations of benzene and toluene in our data were highly intercorrelated in air and blood, with both correlations approaching one [45], disabling us from testing for confounding by toluene or to assess a possible modulating effect of toluene. The urinary concentration of t,t-MA was not significantly different between tank workers and referents in the present study, supported by the poor correlation between environmental benzene and urinary t,t-MA previously reported from a pooled dataset of petroleum workers that also included the present tank workers [16]. There is some conflicting evidence in respect to whether t,t-MA is a reliable marker at sub-ppm levels [46,47]. However, results from numerous studies measuring both urinary benzene and t,t-MA in subjects with mean benzene exposures below 0.1 ppm have repeatedly reported that urinary benzene is a more valid biomarker at the exposure levels that are relevant for our study population and the general population [16,46,48,49,50]. PAH is reported to be an immunosuppressive agent that alters both humoral (B-cell mediated) and cellular (T-cell mediated) immune responses [51], and it may also be present in the tank atmosphere. However, tank workers’ urinary concentration of 1-HP was not significantly different between groups, did not change with time, and it was poorly correlated with benzene in the original study population [16]. Hence, the difference in gene expression between tank workers and referents should therefore be interpreted as being related either to benzene or toluene exposure, or the combination of the two. Interaction effects between benzene and toluene metabolism could either weaken or strengthen responses.

The Norwegian and European occupational exposure limit of benzene (OEL), both averaged over an 8-h shift, is 1 ppm, while the American Conference of Governmental Industrial Hygienists (ACGIH) Threshold Limit Value (TLV^®^) is 0.5 ppm [52]. However, the OEL of benzene is on decline, and the Advisory Committee on Safety and Health at Work in the European Union has been recommended to lower the OEL to 0.05 ppm [53]. Hence, these results, together with observations from other studies [7,8,25,26], thus supports the need for reducing the exposure to benzene in occupational settings. The results are of increasing relevance also for the general population, in developing countries reported to be exposed to city-specific average benzene concentrations in the outdoor air of up to 0.02 ppm [54].

## 5. Conclusions

Gene expression patterns in peripheral blood cells from workers that were exposed to benzene at concentrations well below 0.5 ppm benzene differed significantly as compared to non-exposed referents. Changes in expression of the gene pairs CLEC5/ACSL1 and IL6/IL19 clearly separated exposed workers from referents following a three-day work shift. The results were consistent with other reports and they suggest that gene expression analyses in the study of subjects exposed to low benzene exposure are feasible. Multivariate selection of individual genes facilitates analysis in small datasets.

## Figures and Tables

**Table 1:** Demographics and descriptive statistics for exposure to petroleum-derived hydrocarbons by exposure group.

Parameter	Unbalanced		Balanced	
	Tank Workers	Referents	Tank Workers	Referents
Subjects	8	5	4	4
Sex (male/female)	8/0	4/1	4/0	4/0
Age in years (range)	37.6 (27–54)	48.2 (42–59)	43.5 (38–54)	45.5 (42–53)
Current smokers (%)	3 (37.5%)	1 (20%)	0 (0%)	1 (25%)
Former smokers (%)	4 (50%)	2 (40%)	4 (100%)	1 (25%)
Benzene in air (ppm)	AM (SD) = 0.21 (0.17) GM (GSD) = 0.14 (2.9) Range 0.03–0.45	—	AM (SD) = 0.25 (0.16) GM (GSD) = 0.19 (2.6) Range 0.08–0.50	—
Toluene in air (ppm)	AM (SD) = 0.36 (0.23) GM (GSD) = 0.22 (2.6) Range 0.10–0.8	—	AM (SD) = 0.37 (0.22) GM (GSD) = 0.30 (2.5) Range 0.14–0.71	—
Ethylbenzene in air (ppm)	AM (SD) = 0.10 (0.08) GM (GSD) = 0.07 (2.56) Range 0.02 – 0.22	—	AM (SD) = 0.12 (0.08) GM (GSD) = 0.10 (2.29) Range 0.03–0.21	—
Xylene in air, all isomers (ppm)	AM (SD) = 0.54 (0.40) GM (GSD) = 0.42 (2.27) Range 0.13–1.14	—	AM (SD) = 0.63 (0.37) GM (GSD) = 0.53(2.07) Range 0.20–1.04	—
Benzene in blood (nmol/L)	AM (SD) = 19.3 (12) GM (GSD) = 15.4 (2.2) Range 4.0–38.0	AM (SD) = 0.9 (0.2) GM (GSD) = 0.87 (1.4) Range 0.5–1.0	AM (SD) = 22.8 (12.4) GM (GSD) = 19.7 (1.9) Range 8.0–38.0	AM (SD) = 0.9 (0.3) GM (GSD) = 0.84 (1.4) Range 0.5–1.0
Toluene in blood (nmol/L)	AM (SD) = 28.8 (21.6) GM (GSD) = 21.7 (2.3) Range 6.0–73.0	AM (SD) = 0.9 (0.2) GM (GSD) = 0.87 (1.4) Range 0.5–1.0	AM (SD) = 36.5 (27.0) GM (GSD) = 28.0 (2.5) Range 8.0–73.0	AM (SD) = 1.0 (-) GM (GSD) = (-) Range 1.0–1.0
Benzene in urine (nmol/L)	AM (SD) = 71.3 (110) GM (GSD) = 36.6 (2.9) Range 15.0–333.0	AM (SD) = 1.1 (0.8) GM (GSD) = 0.87 (1.4) Range 0.5–2.0	AM (SD) = 121 (146) GM (GSD) = 65.9 (3.7) Range 20.0–333.0	AM (SD) = 0.88 (0.8) GM (GSD) = 0.71 (2.0) Range 0.5–2.0
Toluene in urine (nmol/L)	AM (SD) = 15.5 (5,3) GM (GSD) = 14.38 (1.6) Range 5.0–23.0	AM (SD) = 3.6 (1.7) GM (GSD) = 3.13 (2.0) Range 1.0–5.0	AM (SD) = 18.8 (3.8) GM (GSD) = 18.45 (1.2) Range 14.0–23.0	AM (SD) = 3.3 (1.7) GM (GSD) = 2.78 (2.0) Range 1.0–5.0
t,t-muconic acid (µmol/L)	AM (SD) = 4.2 (4.1) GM (GSD) = 3.0 (1.9) Range 1.6–14.1	AM (SD) = 2.6 (1.5) GM (GSD) = 2.27 (1.7) Range 1.1–5.1	AM (SD) = 5.8 (5.6) GM (GSD) = 4.35 (2.2) Range 2.5–14.1	AM (SD) = 2.7 (1.7) GM (GSD) = 2.35 (1.9) Range 1.1–5.1
1-Hydroxypyrene in urine (1-HP, µg/g creatinine)	AM (SD) = 0.43 (1.1) GM (GSD) = 0.05 (7.0) Range 0.01–3.18	AM (SD) = 0.01 (0.01) GM (GSD) = 0.01 (1.9) Range 0.006–0.036	AM (SD) = 0.8 (1.6) GM (GSD) = 0.09 (13.4) Range 0.007–3.18	AM (SD) = 0.01 (0.01) GM (GSD) = 0.01 (2.1) Range 0.006–0.036

AM = arithmetic mean, SD = standard deviation, GM = geometric mean, GSD = geometric standard deviation. Chemical exposure reported at third day of study (air) and post-shift (biomarkers, t_1_).

## Supplementary Materials

The following are available online at http://www.mdpi.com/1660-4601/15/11/2385/s1, Table S1a. Participant questionnaire in Norwegian (original), Table S1b. Participant questionnaire in English (translated), Table S2. Complete dataset all parameters (except genes), Figure S1. Elastic Net model, fold change of six genes from Schiffman and coworkers, on age balanced workers using exposure group as the response Top: Time point 1. Bottom: Time point 2. Left: Cross-validation curves (red) with standard deviation (grey) as the number of selected genes decreases. Right: Coefficient curves for each transcript with increasing number of variables. Table S3. Gene IDs of six genes selected by Schiffman as markers for low dose benzene exposure in all workers. Figure S2. PCA model of all six genes with fold change selected by Elastic Net at time point 2 using six Schiffman genes as input and exposed vs. unexposed as response. Figure S3. Individual plots of the expression as fold change of all six Schiffman genes in all workers Figure S4. Elastic net, PCA model and individual gene plots of twelve genes selected by Elastic Net from 30 genes tested by Schiffman in age balanced data with fold change at time 2 using exposed vs. unexposed as the response. Table S4 Gene IDs of twelve genes selected by Elastic Net from 30 genes tested by Schiffman in age balanced data with fold change at time 2 using exposed vs. unexposed as the response. Figure S5 Elastic Net model, fold change of genes belonging to the Jak-STAT pathway on age balanced workers using exposure group as the response. Top: Time point 1. Bottom: Time point 2. Left: Cross-validation curves (red) with standard deviation (grey) as the number of selected genes decreases. Right: Coefficient curves for each transcript with increasing number of variables. Table S5. Gene IDs of sixteen genes selected by Elastic net at time point 2 from the Jak-STAT pathway in age balanced data with fold change using exposed vs. unexposed as response. Figure S6 Individual plots of the expression of sixteen genes selected by Elastic net at time point 2 from the Jak-STAT pathway in age balanced data with fold change using exposed vs. unexposed as response. Table S6. Gene IDs of 8 genes selected by Elastic Net from 30 genes tested by Schiffman at time point 0 and two at time point in age balanced data without fold change using exposed vs. unexposed as the response. Figure S7: Pairwise plots of selected marker genes (CLEC5A/ACSL1, IL6/IL19, IL6/SOCS1). Page 1: With fold change at time 2. Page 2: Without fold change at time 0. Table S7: Gene IDs of ten genes selected by Elastic net at time point 0 and two at time point 2 from the Jak-STAT pathway in age balanced data without fold change using exposed vs. unexposed as response. Figure S8 Elastic Net, PCA models and individual gene plots of eight genes selected from 30 genes tested by Schiffman at time point 0 and two at time point 1 in age balanced data without fold change using exposed vs. unexposed as the response. Figure S9. Elastic Net, PCA models and individual gene plots of ten genes selected from genes belonging to the Jak-STAT pathway at time point 0 and two at time point 2 in age balanced data without fold change using exposed vs. unexposed as the response. Figure S10. Time series plots of average gene expression without fold change of 20 selected marker genes in referents vs. exposed workers. Figure S11. Confidence intervals of the difference in average gene expression between referent and exposed workers of 20 selected marker genes in data without fold change (page 1) and with fold change (page 2). Other files: running scripts for R for all models and graphs.
