# Supplementary material for: Gene Expression Response in Peripheral Blood Cells of Petroleum Workers Exposed to Sub-Ppm Benzene Levels
Source: Int J Environ Res Public Health. 2018 Oct 27;15(11):2385. doi: 10.3390/ijerph15112385 (PMC6266895; doi:10.3390/ijerph15112385)
Supplement: Supplementary file 1 [file ijerph-15-02385-s001.zip › ijerph-344087-SI/Suppl info corrected/S10 Figure.pdf]

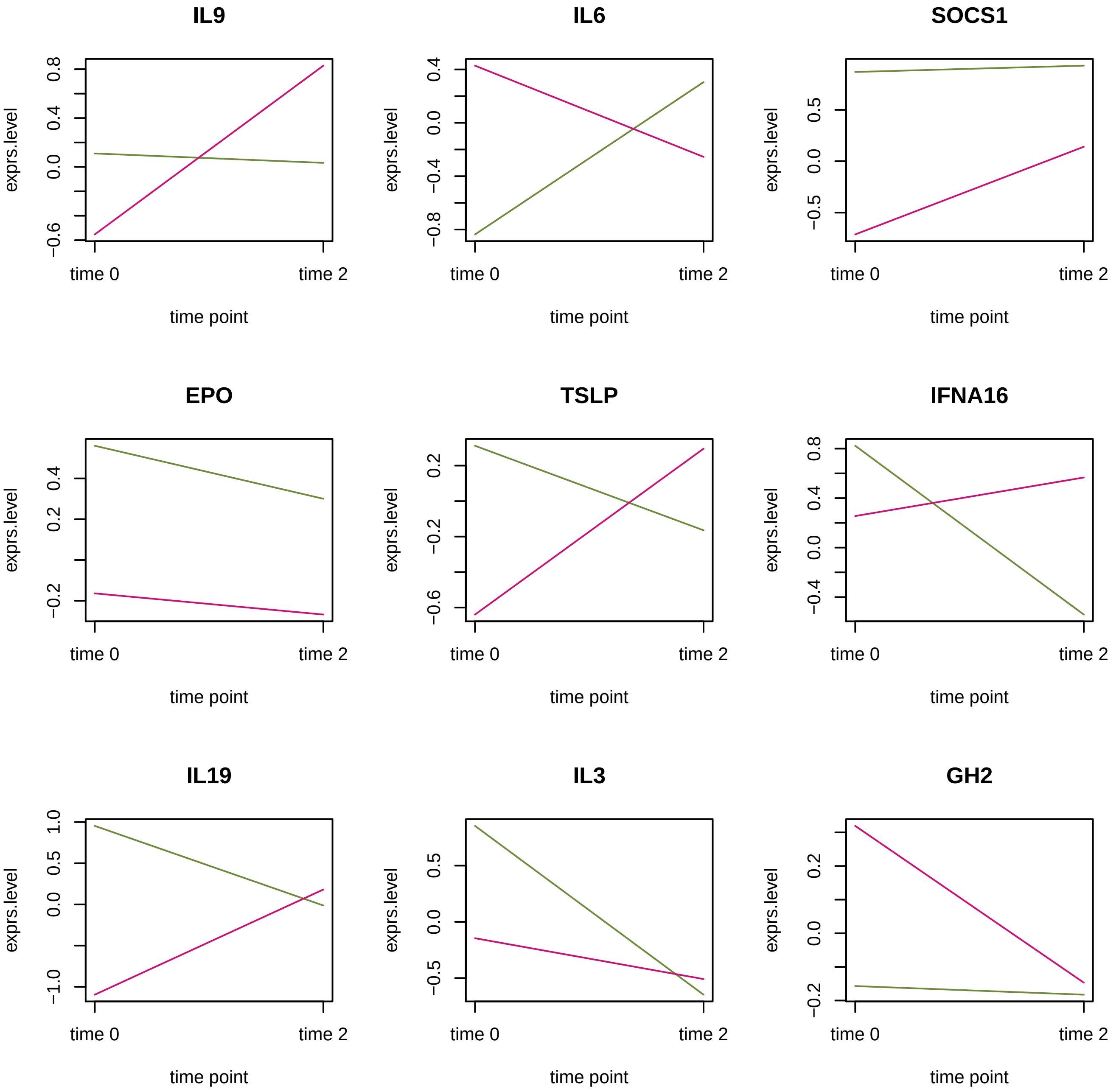
IL9
IL6
SOCS1
EPO
TSLP
IFNA16
IL19
IL3
GH2
exprs.level
time point
time 0
time 2
−0.6
0.0
0.4
0.8
−0.8
−0.4
0.4
−0.5
0.5
−0.2
0.2
1.0
−1.0

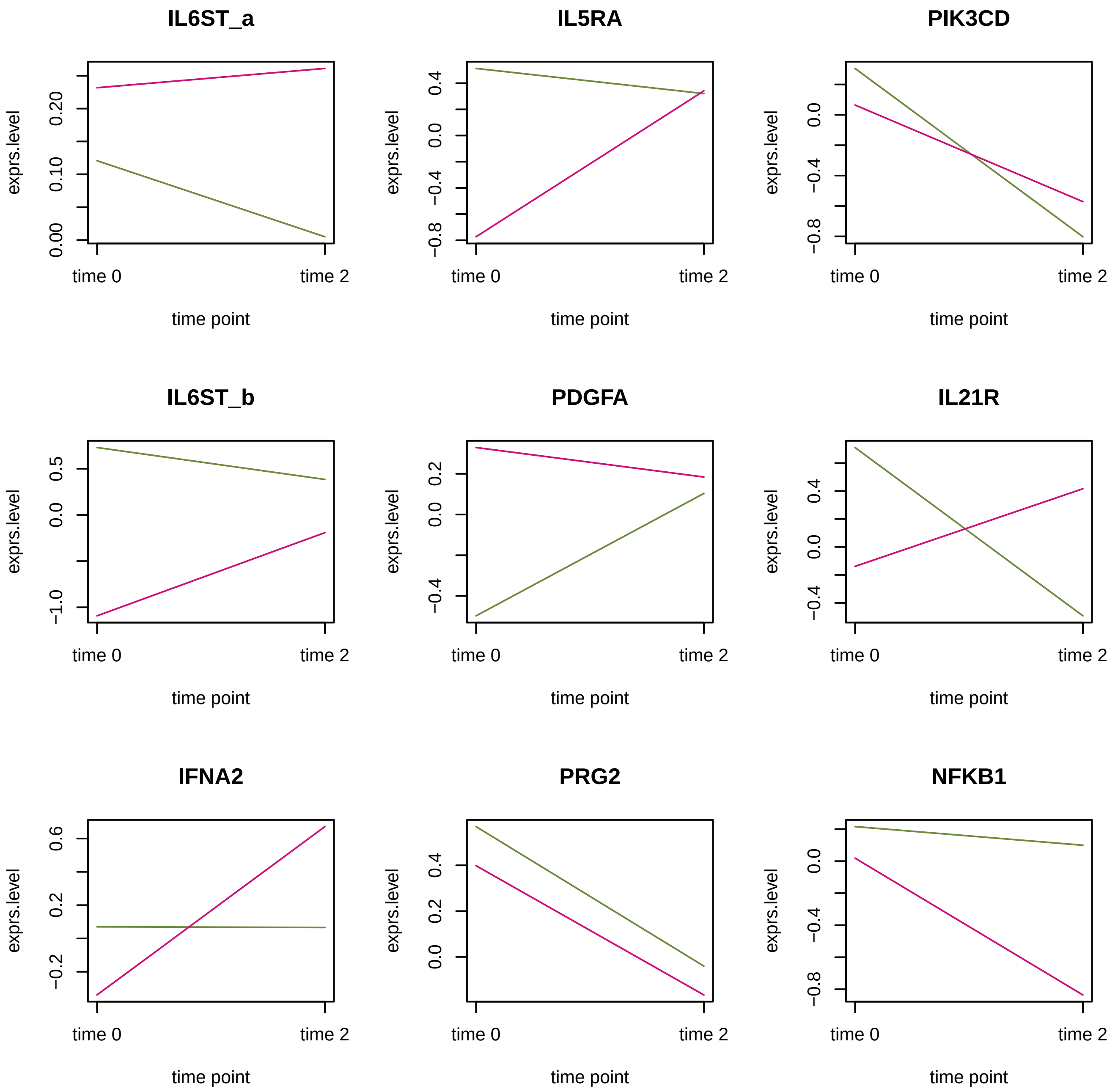
IL6ST_a
0.00
0.10
0.20
exprs.level
time 0
time 2
time point
IL5RA
−0.8
−0.4
0.0
0.4
exprs.level
time 0
time 2
time point
PIK3CD
−0.8
−0.4
0.0
exprs.level
time 0
time 2
time point
IL6ST_b
−1.0
0.0
0.5
exprs.level
time 0
time 2
time point
PDGFA
−0.4
0.0
0.2
exprs.level
time 0
time 2
time point
IL21R
−0.4
0.0
0.4
exprs.level
time 0
time 2
time point
IFNA2
−0.2
0.2
0.6
exprs.level
time 0
time 2
time point
PRG2
0.0
0.2
0.4
exprs.level
time 0
time 2
time point
NFKB1
−0.8
−0.4
0.0
exprs.level
time 0
time 2
time point

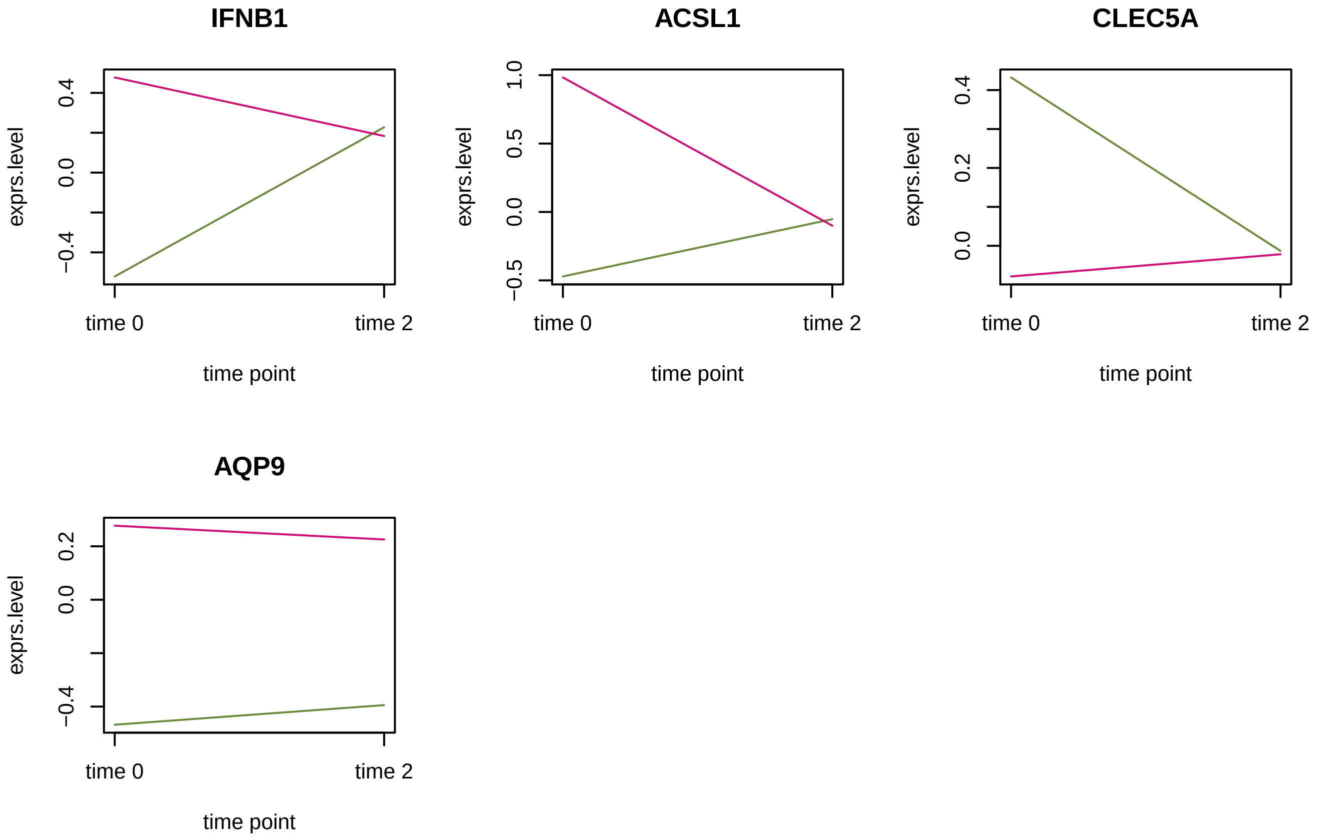
IFNB1
ACSL1
CLEC5A
AQP9
exprs.level
time 0
time 2
time point
0.4
0.0
−0.4
1.0
0.5
0.0
−0.5
0.2
0.0
0.4
0.2
0.0
0.0
−0.4
