# Supplementary material for: Gene Expression Response in Peripheral Blood Cells of Petroleum Workers Exposed to Sub-Ppm Benzene Levels
Source: Int J Environ Res Public Health. 2018 Oct 27;15(11):2385. doi: 10.3390/ijerph15112385 (PMC6266895; doi:10.3390/ijerph15112385)
Supplement: Supplementary file 1 [file ijerph-15-02385-s001.zip › ijerph-344087-SI/Suppl info corrected/S4 Figure.pdf]

China 33 genes with fold change

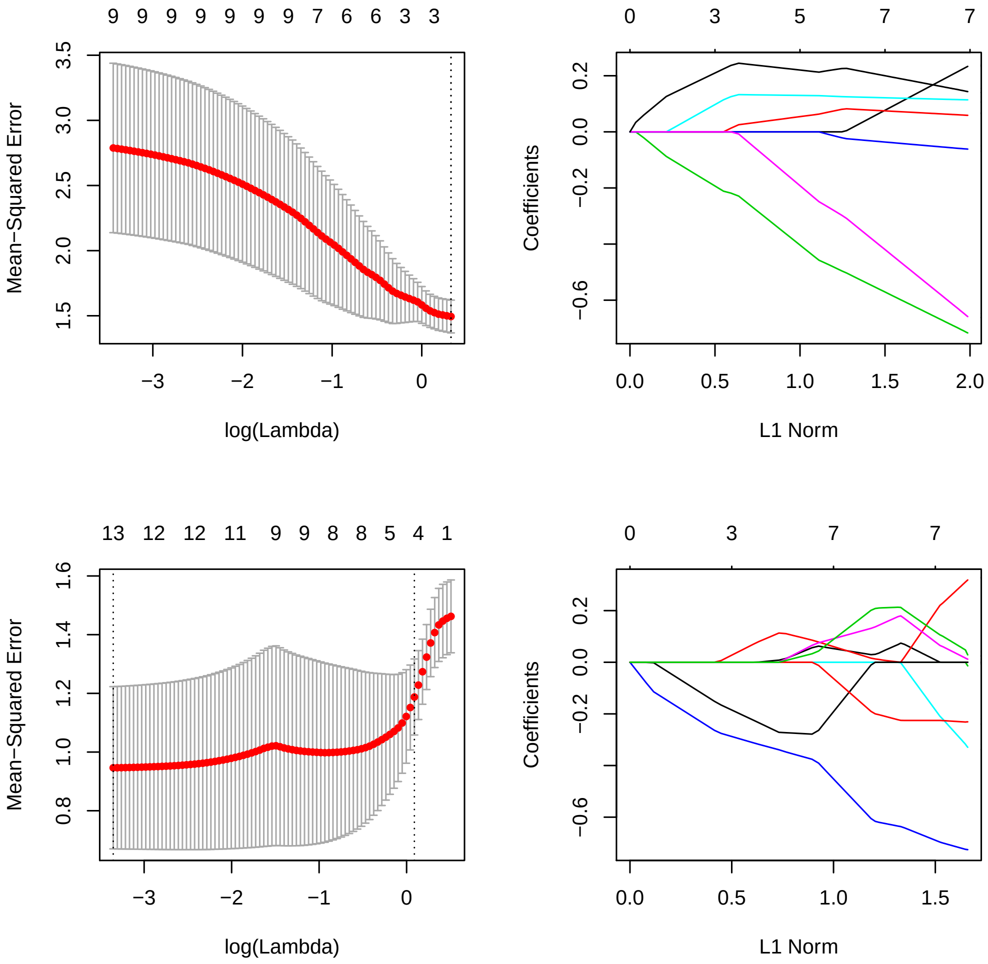

5 genes selected at time 2

China 33 genes with fold change

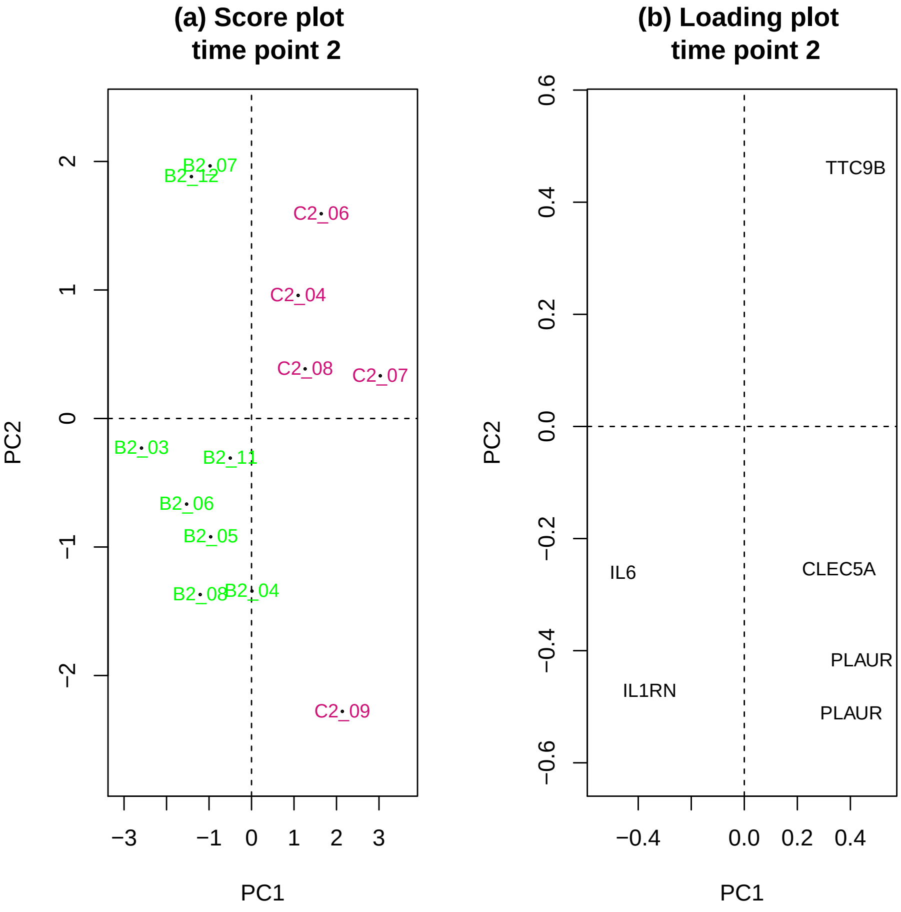

5 genes selected at time 2
