# Supplementary material for: Gene Expression Response in Peripheral Blood Cells of Petroleum Workers Exposed to Sub-Ppm Benzene Levels
Source: Int J Environ Res Public Health. 2018 Oct 27;15(11):2385. doi: 10.3390/ijerph15112385 (PMC6266895; doi:10.3390/ijerph15112385)

The 16 selected Jak-STAT fold change genes at time 2

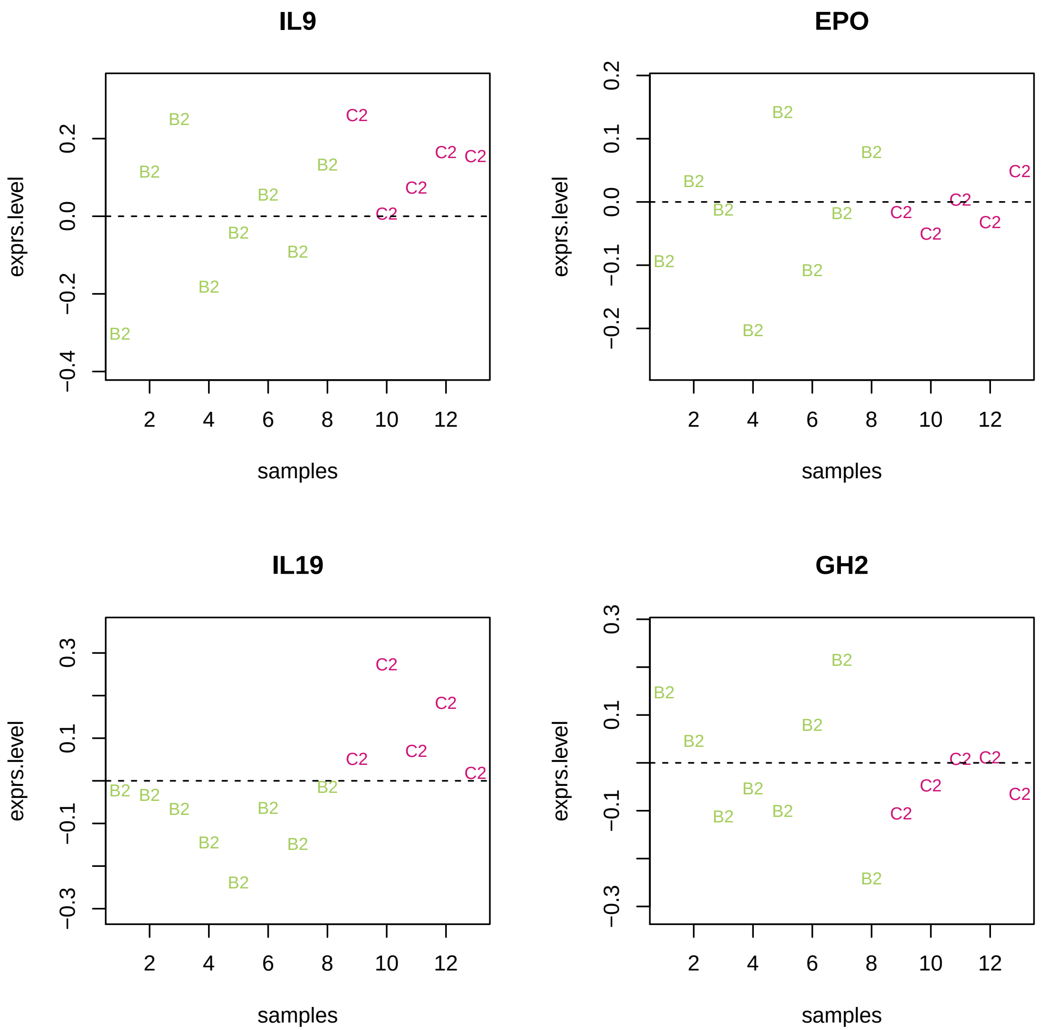

The 16 selected Jak-STAT fold change genes at time 2

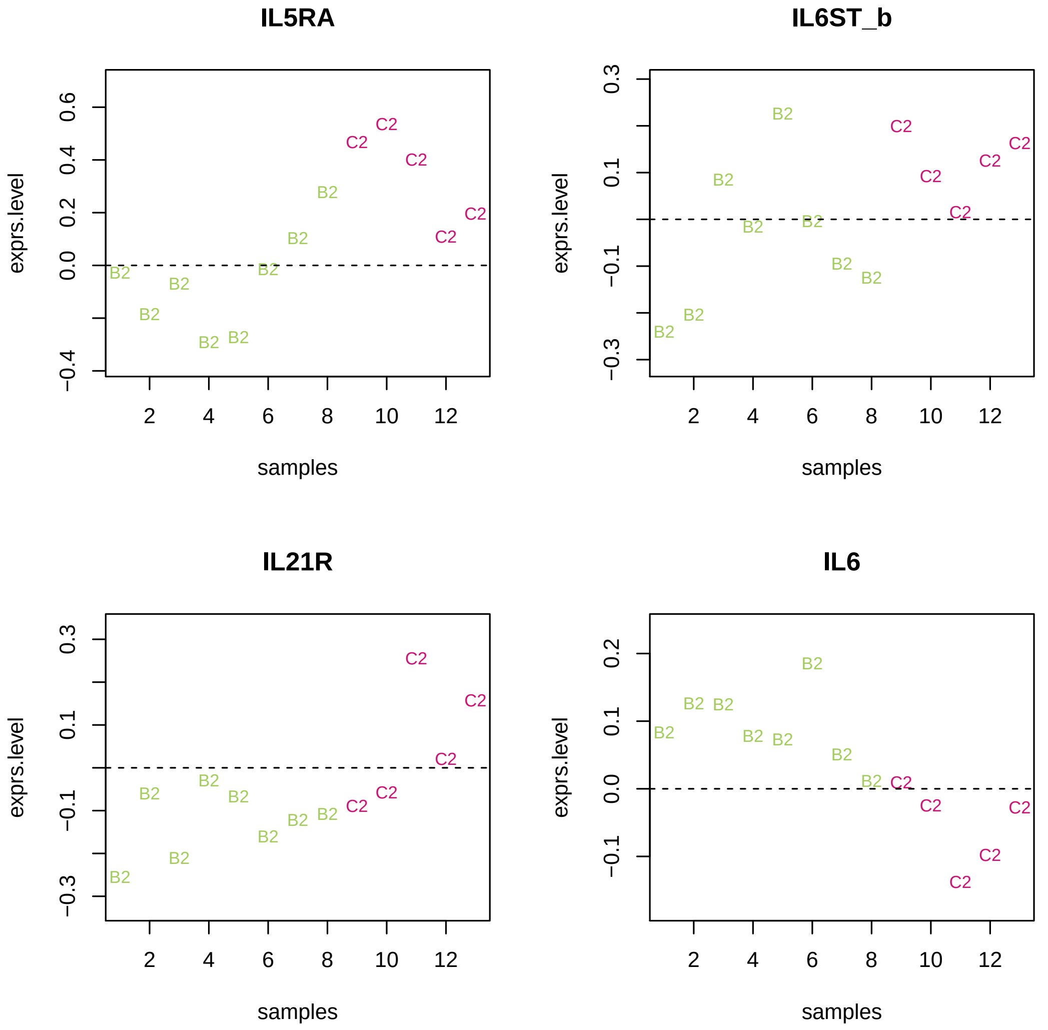

The 16 selected Jak-STAT fold change genes at time 2

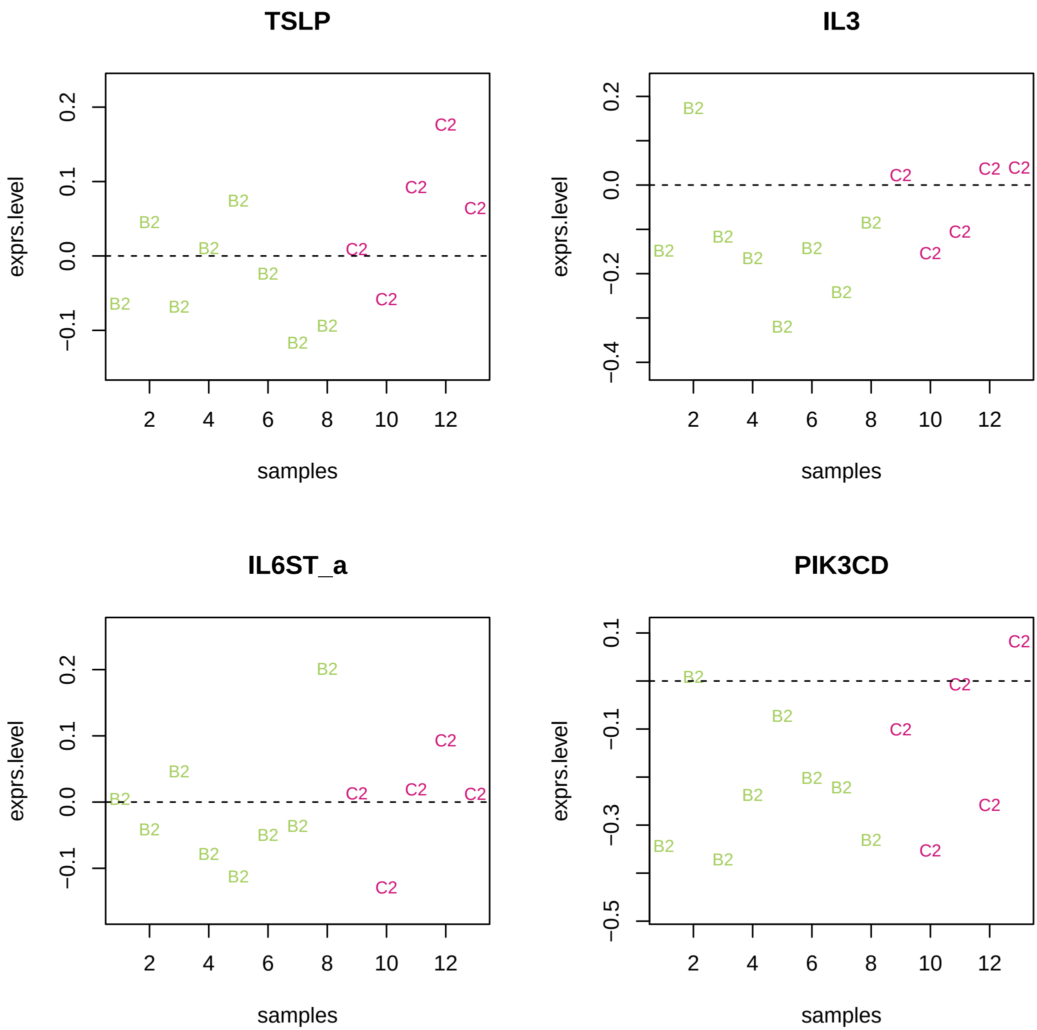

The 16 selected Jak-STAT fold change genes at time 2

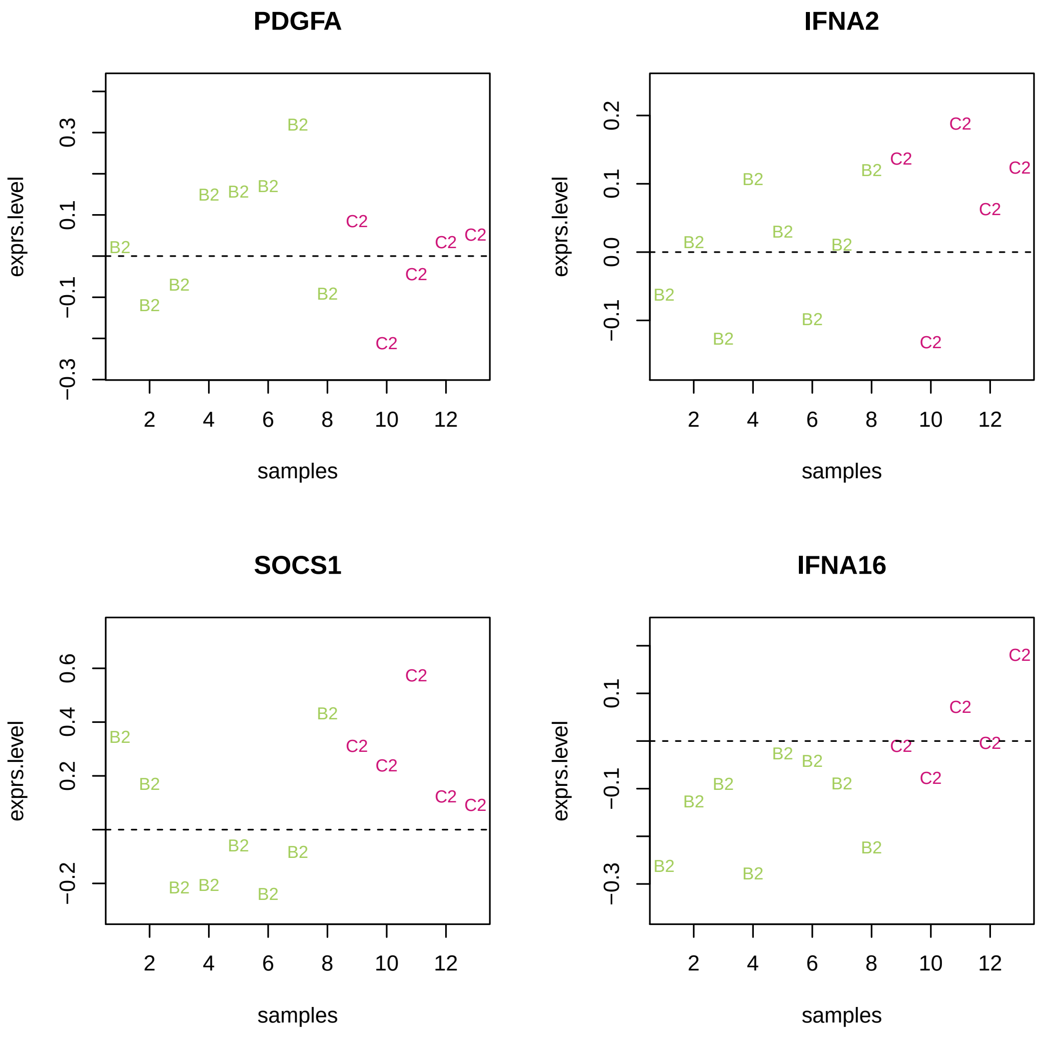

Supplement: Supplementary file 1 [file ijerph-15-02385-s001.zip › ijerph-344087-SI/Suppl info corrected/S6 Figure.pdf]
