# Supplementary material for: Gene Expression Response in Peripheral Blood Cells of Petroleum Workers Exposed to Sub-Ppm Benzene Levels
Source: Int J Environ Res Public Health. 2018 Oct 27;15(11):2385. doi: 10.3390/ijerph15112385 (PMC6266895; doi:10.3390/ijerph15112385)
Supplement: Supplementary file 1 [file ijerph-15-02385-s001.zip › ijerph-344087-SI/Suppl info corrected/S7 Figure.pdf]

Gene plots of best pairwise markers

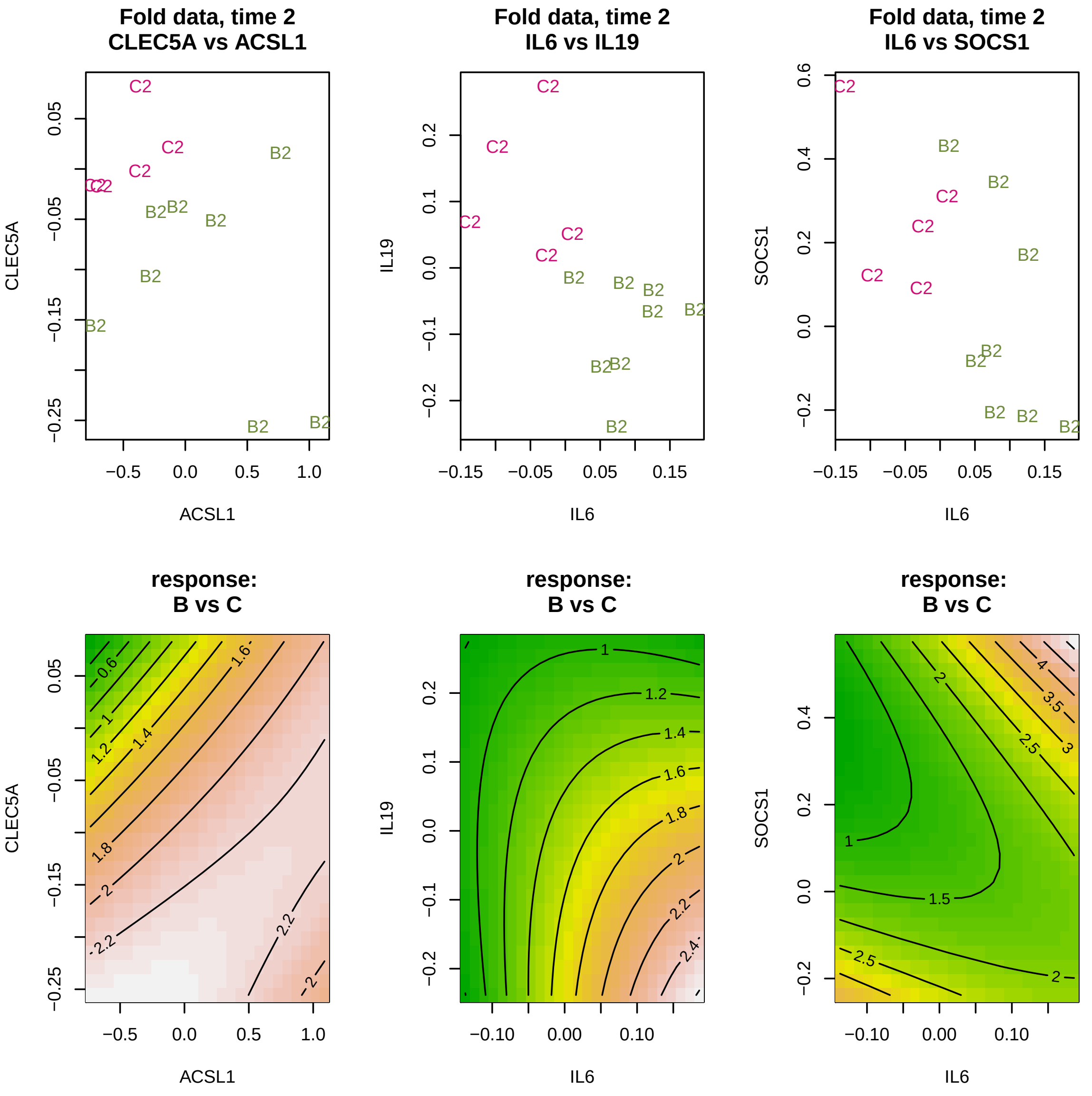

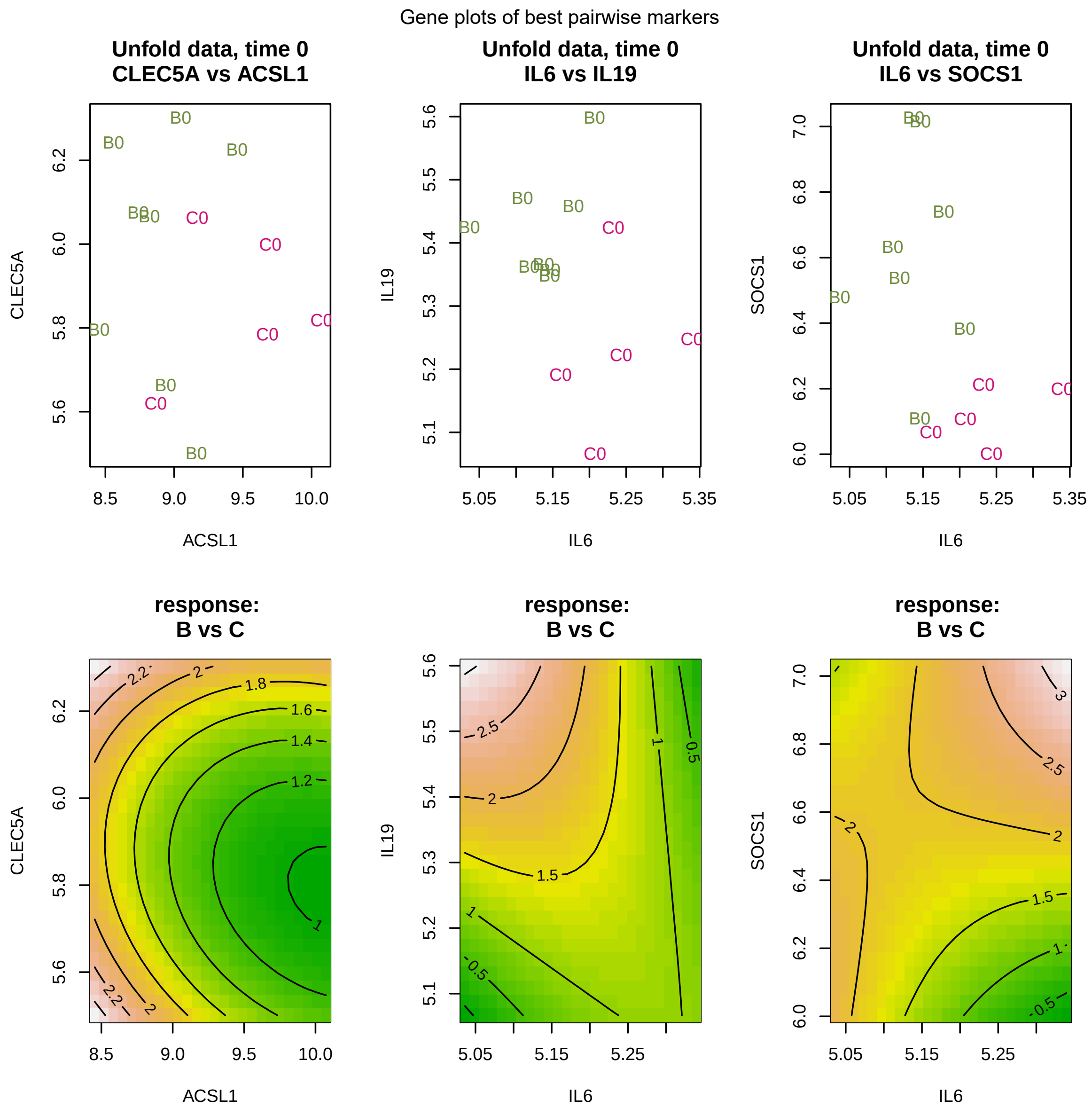
Gene plots of best pairwise markers
Unfold data, time 0
CLEC5A vs ACSL1
Unfold data, time 0
IL6 vs IL19
Unfold data, time 0
IL6 vs SOCS1
response:
B vs C
response:
B vs C
response:
B vs C
CLEC5A
ACSL1
IL19
IL6
SOCS1
B0
C0
