# Supplementary material for: Gene Expression Response in Peripheral Blood Cells of Petroleum Workers Exposed to Sub-Ppm Benzene Levels
Source: Int J Environ Res Public Health. 2018 Oct 27;15(11):2385. doi: 10.3390/ijerph15112385 (PMC6266895; doi:10.3390/ijerph15112385)
Supplement: Supplementary file 1 [file ijerph-15-02385-s001.zip › ijerph-344087-SI/Suppl info corrected/S8 Figure.pdf]

China 30 genes no fold change

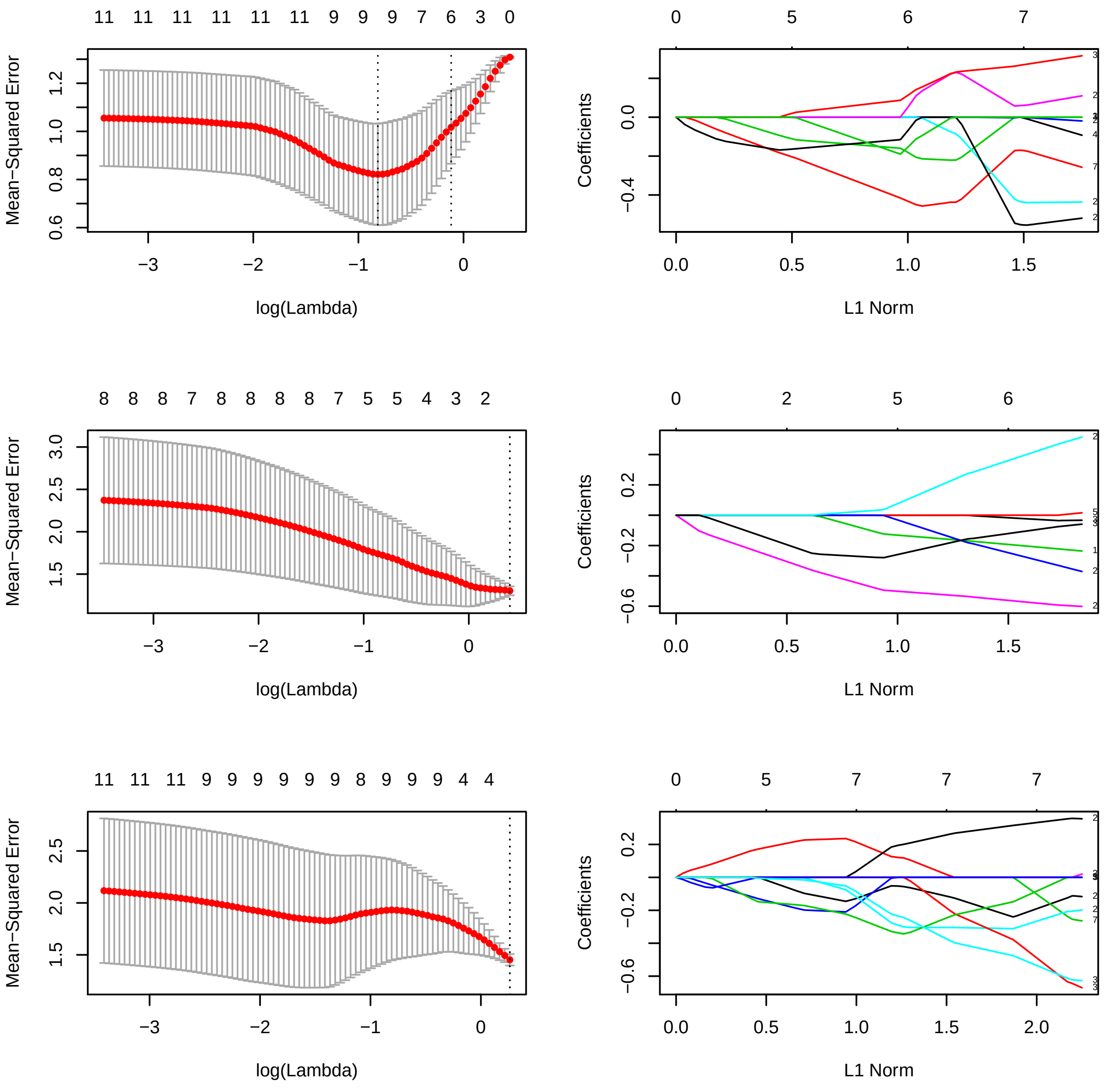

9 genes selected at time 0

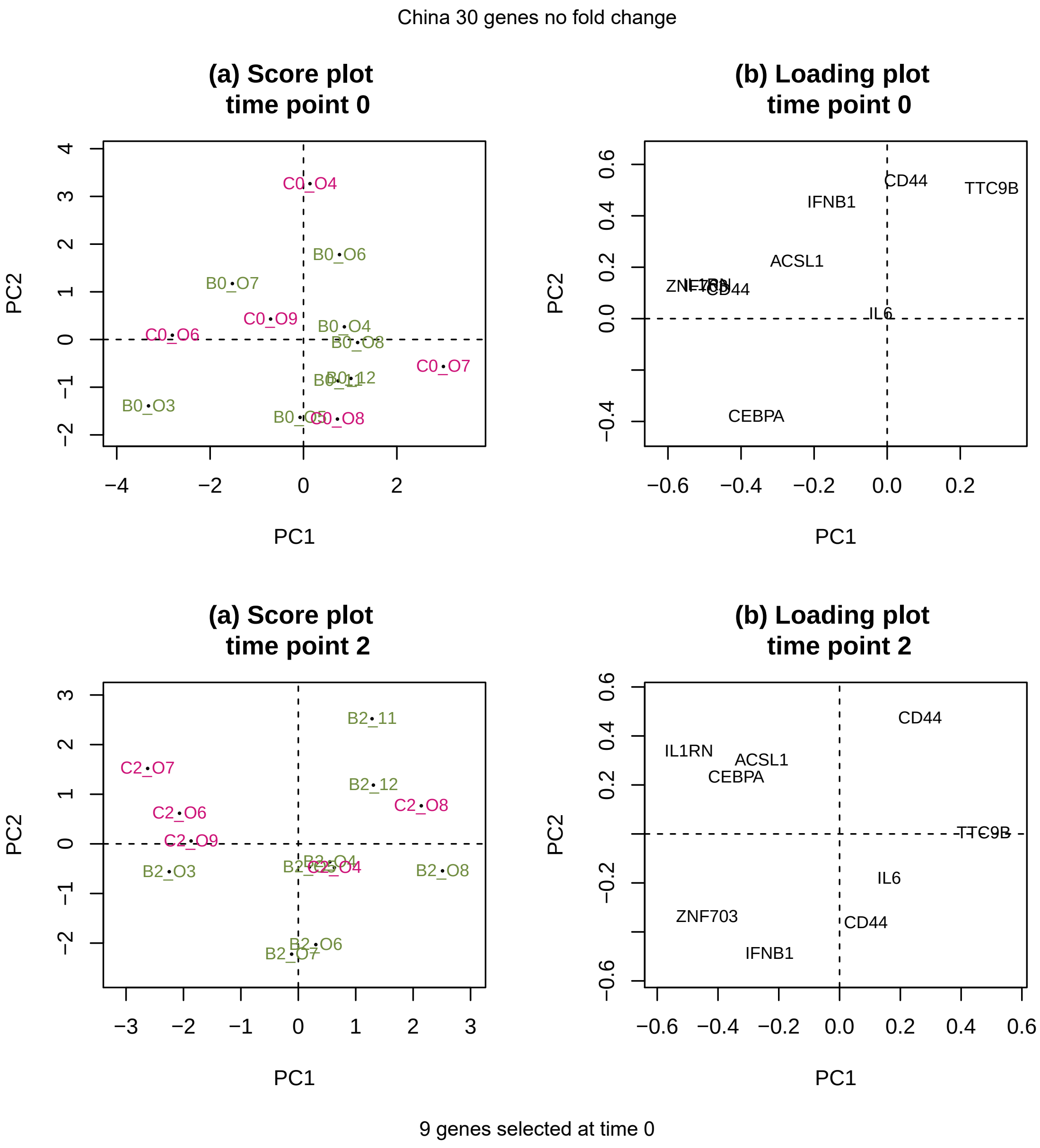
China 30 genes no fold change
(a) Score plot
time point 0
PC2
PC1
C0_O4
B0_O6
B0_O7
C0_O9
B0_O4
C0_O6
B0_O8
C0_O7
B0_12
B0_O3
C0_O8
(b) Loading plot
time point 0
PC2
PC1
CD44
TTC9B
IFNB1
ACSL1
IL6
CEBPA
(a) Score plot
time point 2
PC2
PC1
B2_11
C2_O7
B2_12
C2_O8
C2_O6
C2_O9
B2_O3
B2_O8
B2_O6
B2_O7
(b) Loading plot
time point 2
PC2
PC1
CD44
IL1RN
ACSL1
CEBPA
TTC9B
IL6
ZNF703
CD44
IFNB1
9 genes selected at time 0
