# Supplementary material for: Gene Expression Response in Peripheral Blood Cells of Petroleum Workers Exposed to Sub-Ppm Benzene Levels
Source: Int J Environ Res Public Health. 2018 Oct 27;15(11):2385. doi: 10.3390/ijerph15112385 (PMC6266895; doi:10.3390/ijerph15112385)

Average gene expression without fold change with time in genes selected by Elastic Net in data with fold change

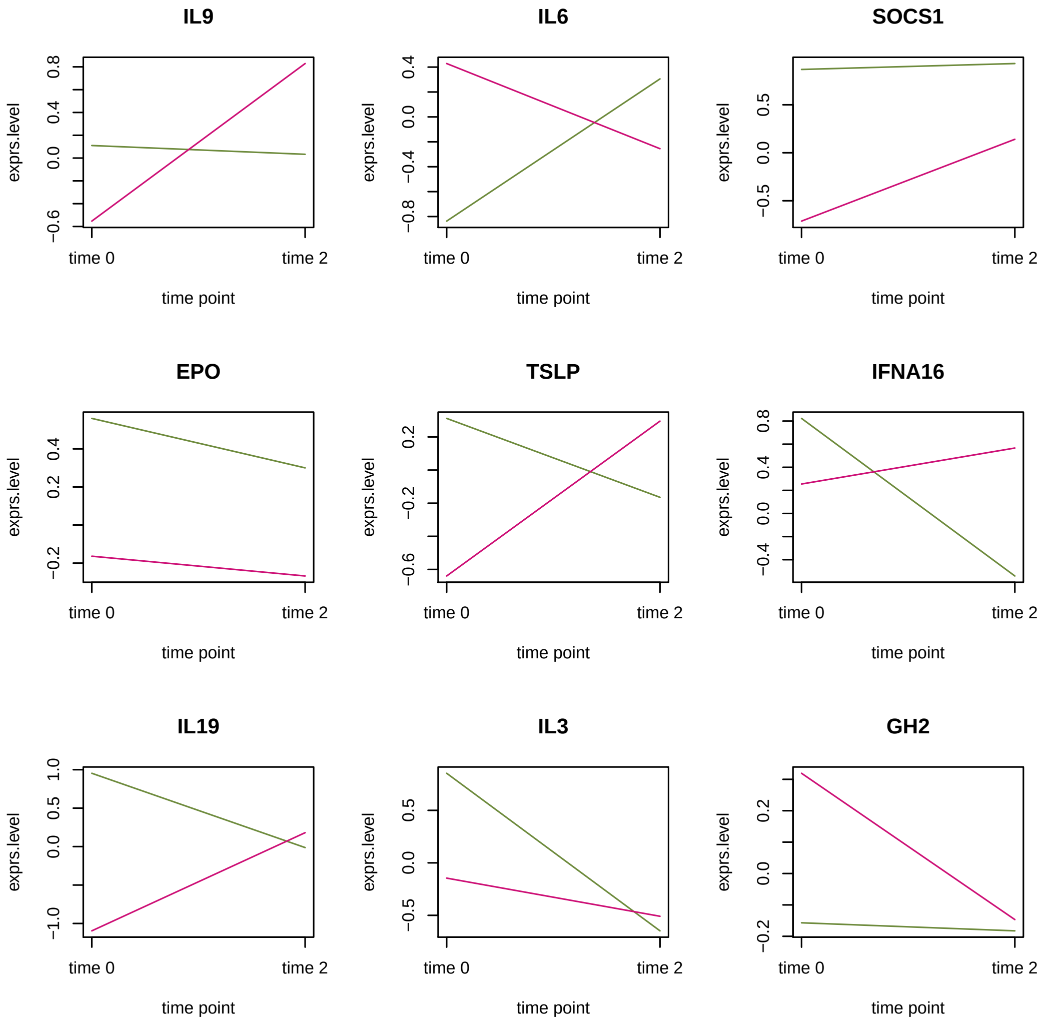

Green: Exposed workers, Pink: Referents

Average gene expression without fold change with time in 20 genes selected by Elastic Net in data with fold change

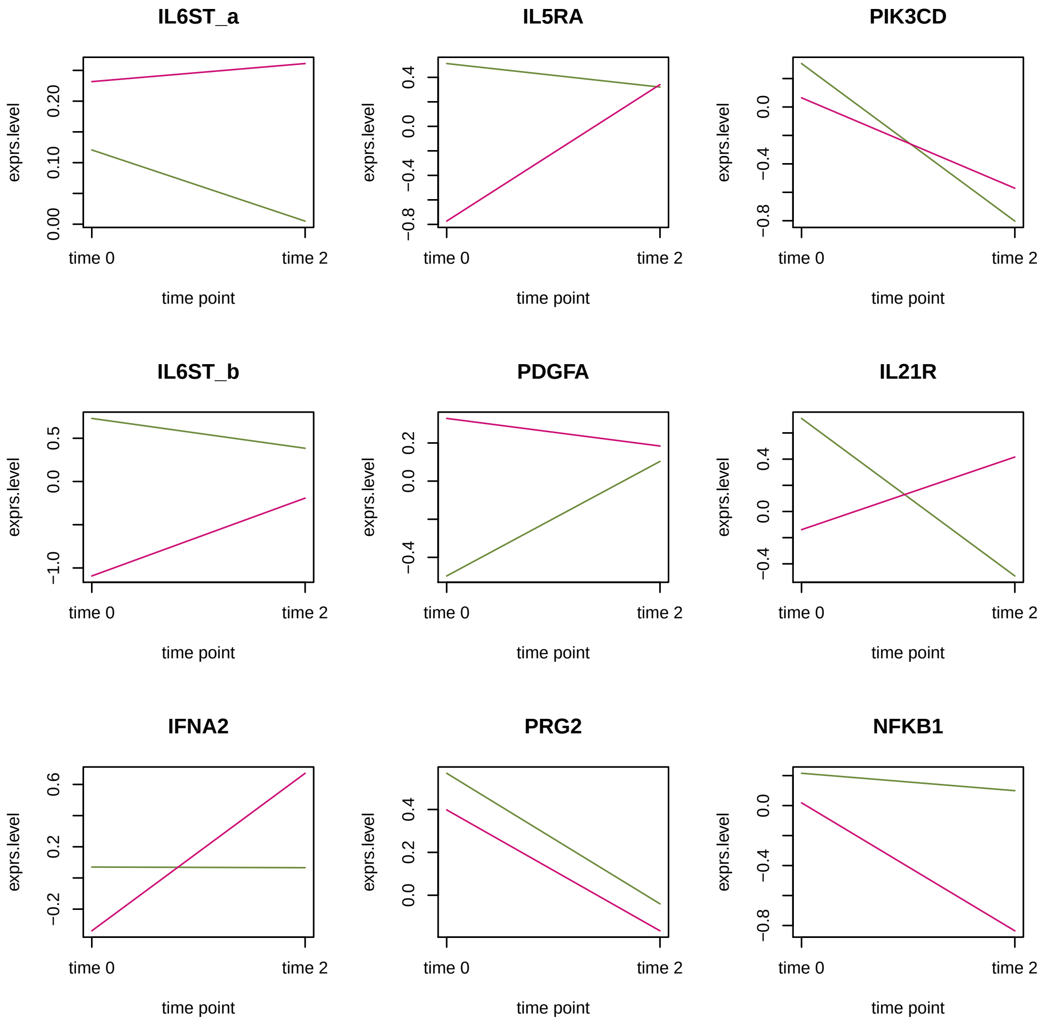

Green: Exposed workers, Pink: Referents

Average gene expression without fold change with time in 20 genes selected by Elastic Net in data with fold change

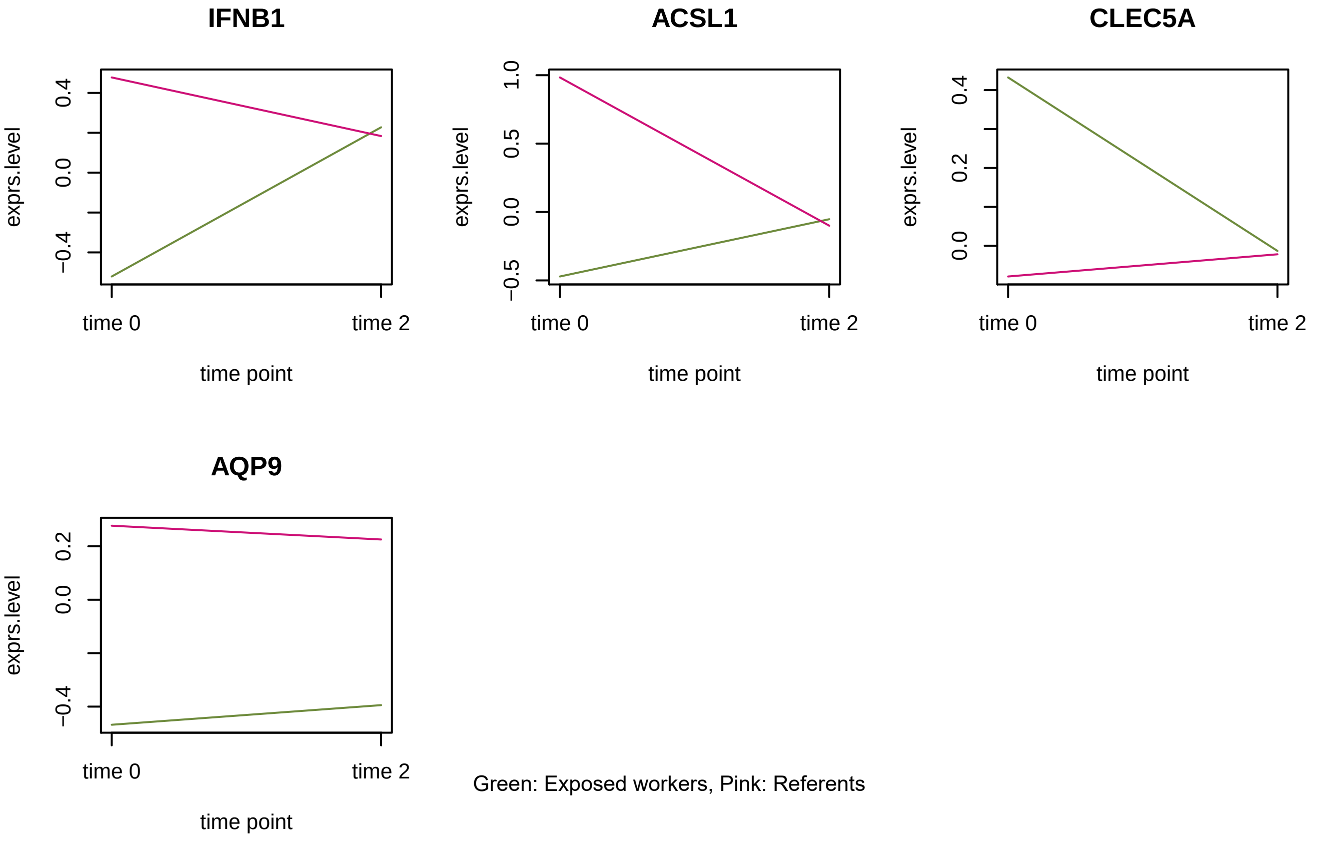

Supplement: Supplementary file 1 [file ijerph-15-02385-s001.zip › ijerph-344087-SI/Supplementary Information Nu/S10 Figure.pdf]
