# Supplementary material for: Gene Expression Response in Peripheral Blood Cells of Petroleum Workers Exposed to Sub-Ppm Benzene Levels
Source: Int J Environ Res Public Health. 2018 Oct 27;15(11):2385. doi: 10.3390/ijerph15112385 (PMC6266895; doi:10.3390/ijerph15112385)
Supplement: Supplementary file 1 [file ijerph-15-02385-s001.zip › ijerph-344087-SI/Supplementary Information Nu/S4 Figure.pdf]

Analysis of all 30 China (Schiffman) marker genes with fold change

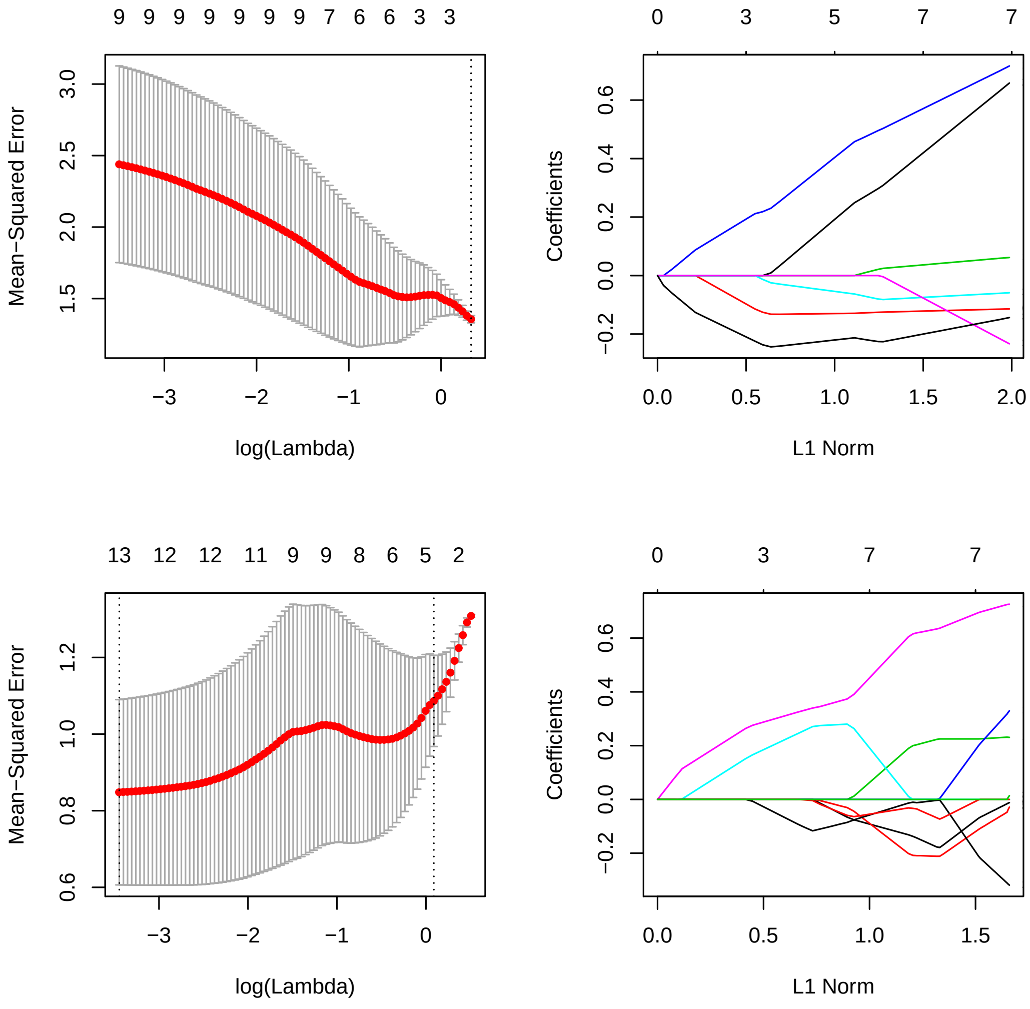

Elastic net results at time1 (top) and 2 (bottom).
Left: Cross validation curves. Right: Coefficients for individual transcripts

Analysis of all 30 China (Schiffman) marker genes with fold change

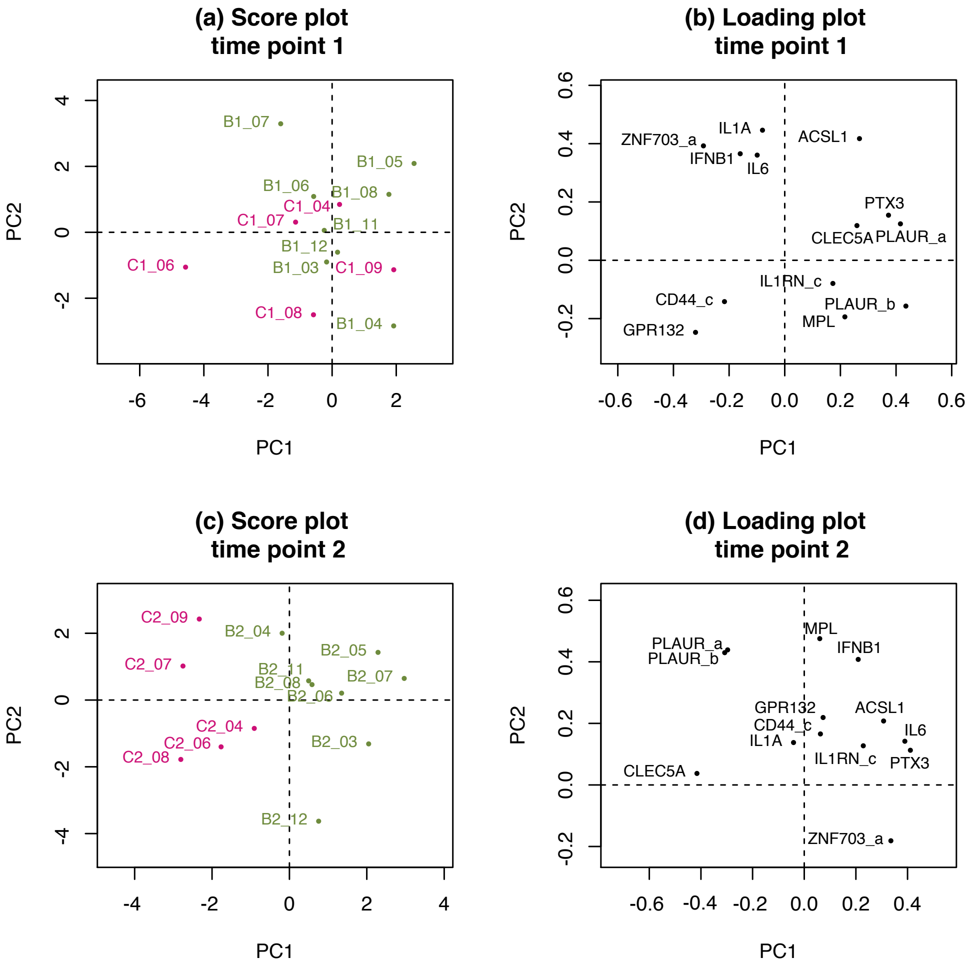

PCA of transcripts selected by Elastic Net at time 2 plotted at both time 1 (top) and time 2 (bottom)

Analysis of all 30 China (Schiffman) marker genes with fold change

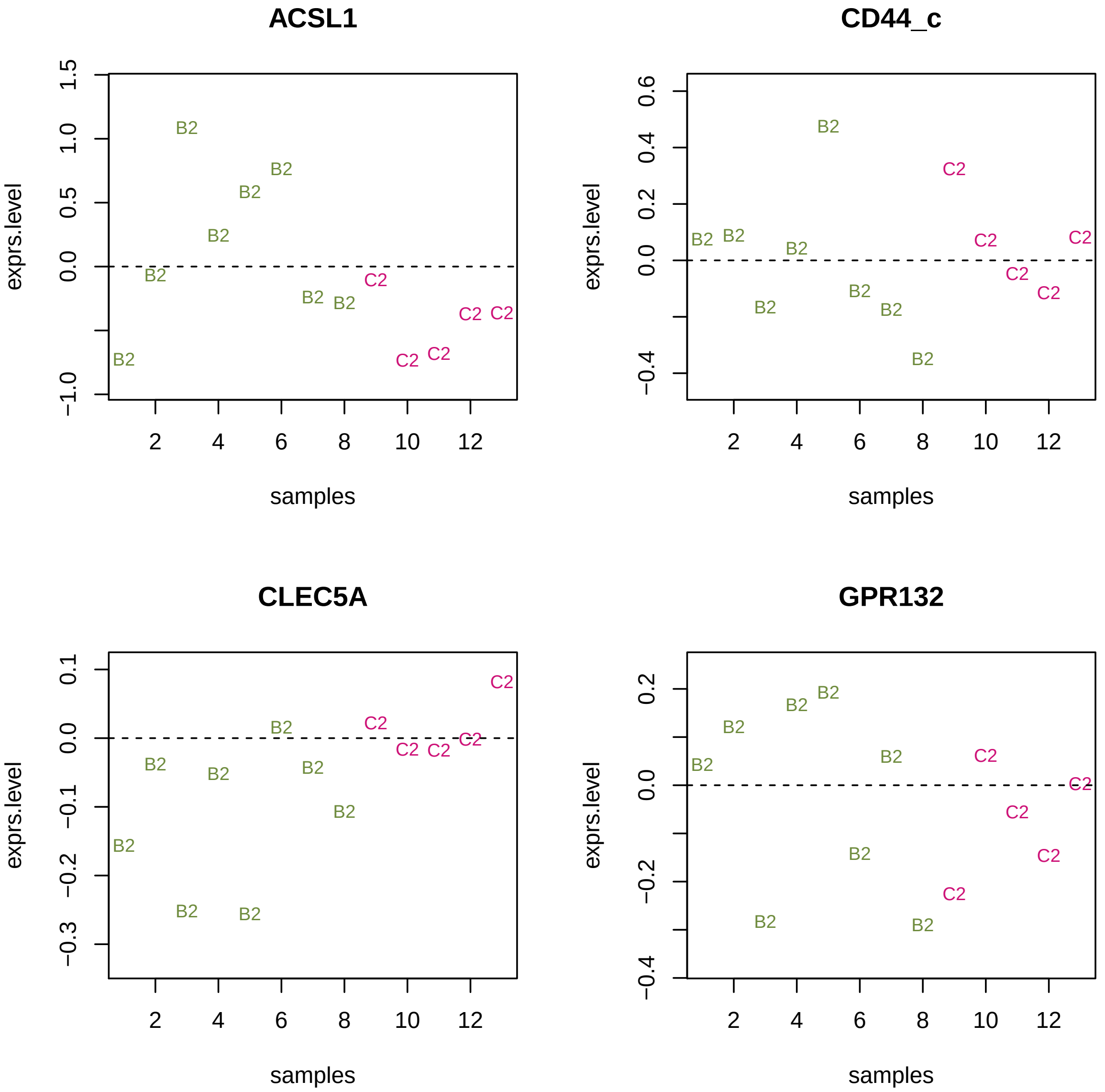

Plots of expression at time 2 in all workers
of individual transcripts selected by Elastic Net at time 2

Analysis of all 30 China (Schiffman) marker genes with fold change

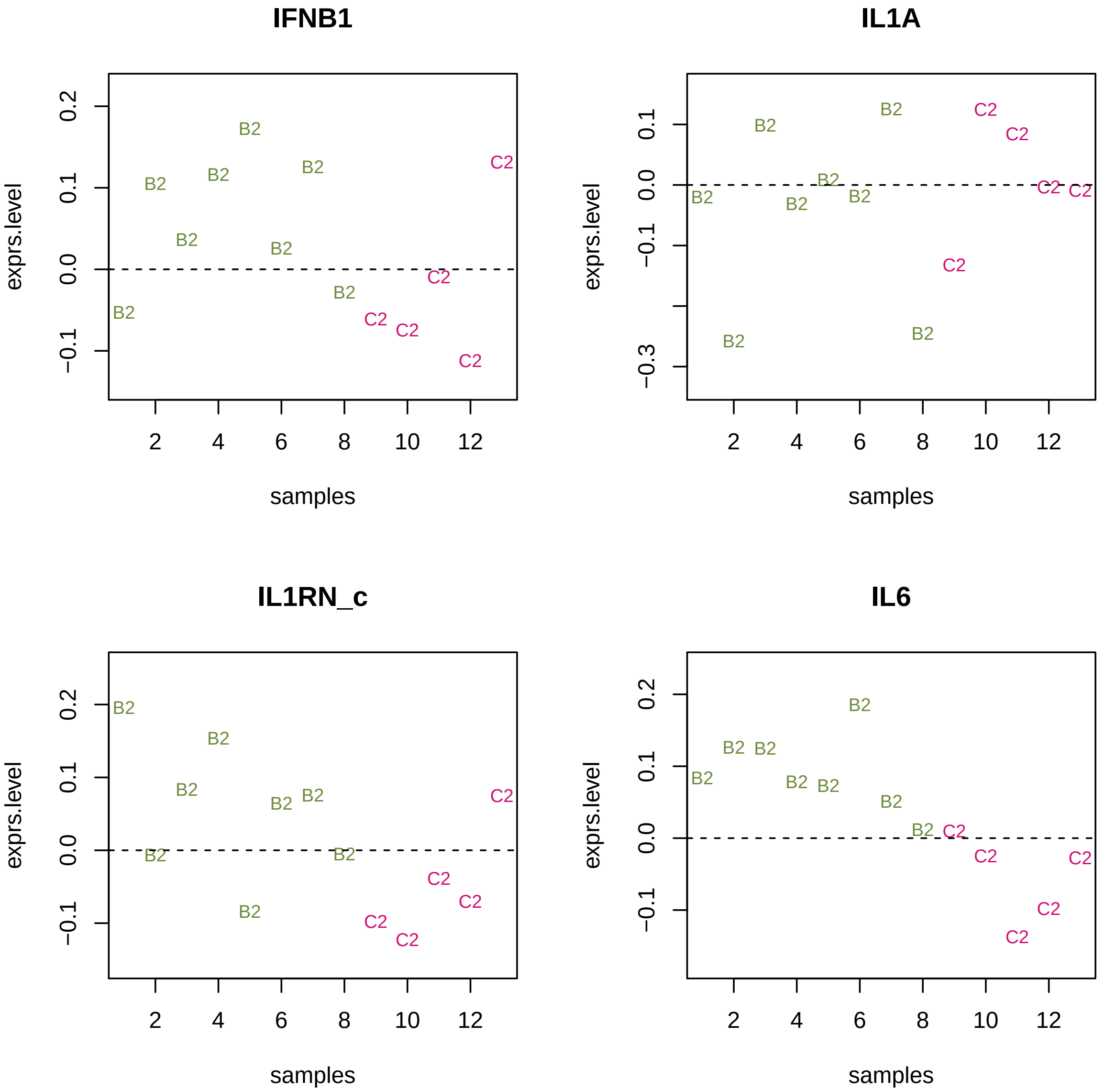

Plots of expression at time 2 in all workers
of individual transcripts selected by Elastic Net at time 2

Analysis of all 30 China (Schiffman) marker genes with fold change

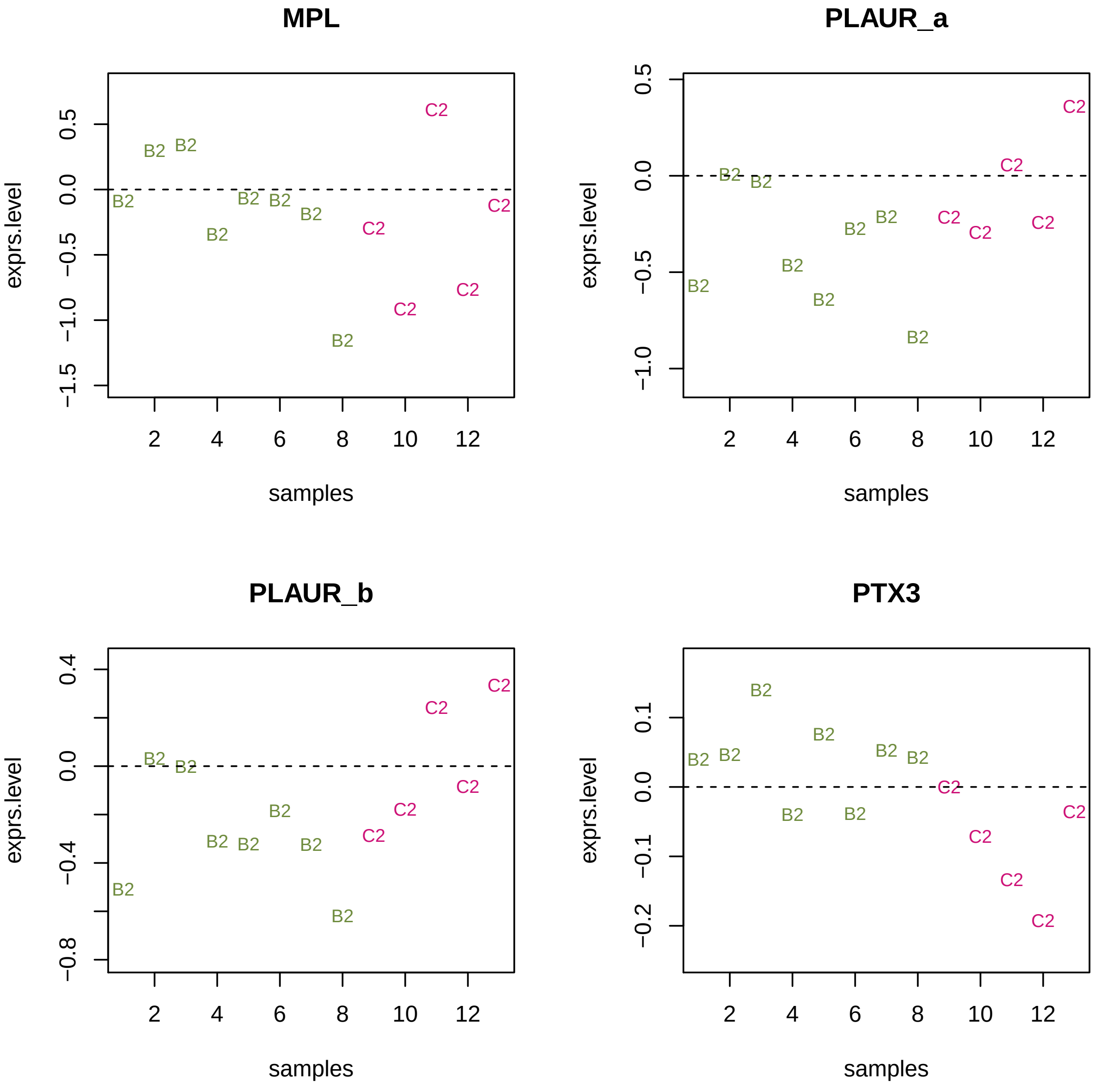

Plots of expression at time 2 in all workers
of individual transcripts selected by Elastic Net at time 2

Analysis of all 30 China (Schiffman) marker genes with fold change

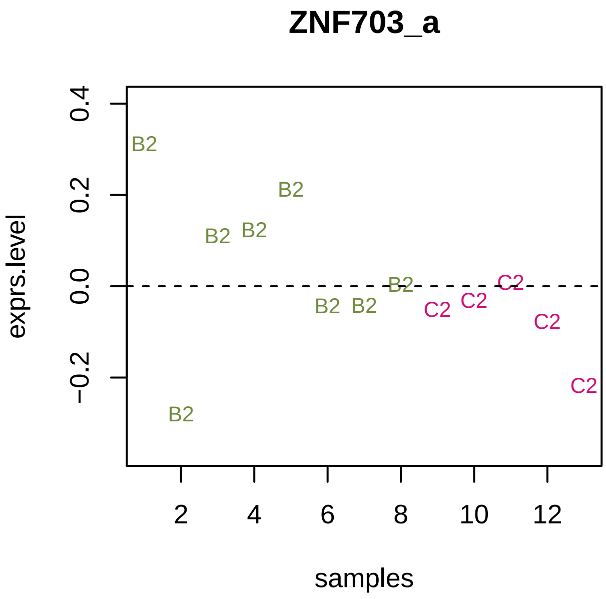

Plots of expression at time 2 in all workers
of individual transcripts selected by Elastic Net at time 2
