# Supplementary material for: Gene Expression Response in Peripheral Blood Cells of Petroleum Workers Exposed to Sub-Ppm Benzene Levels
Source: Int J Environ Res Public Health. 2018 Oct 27;15(11):2385. doi: 10.3390/ijerph15112385 (PMC6266895; doi:10.3390/ijerph15112385)
Supplement: Supplementary file 1 [file ijerph-15-02385-s001.zip › ijerph-344087-SI/Supplementary Information Nu/S6 Figure.pdf]

Analysis of Jak-STAT pathway genes with fold change

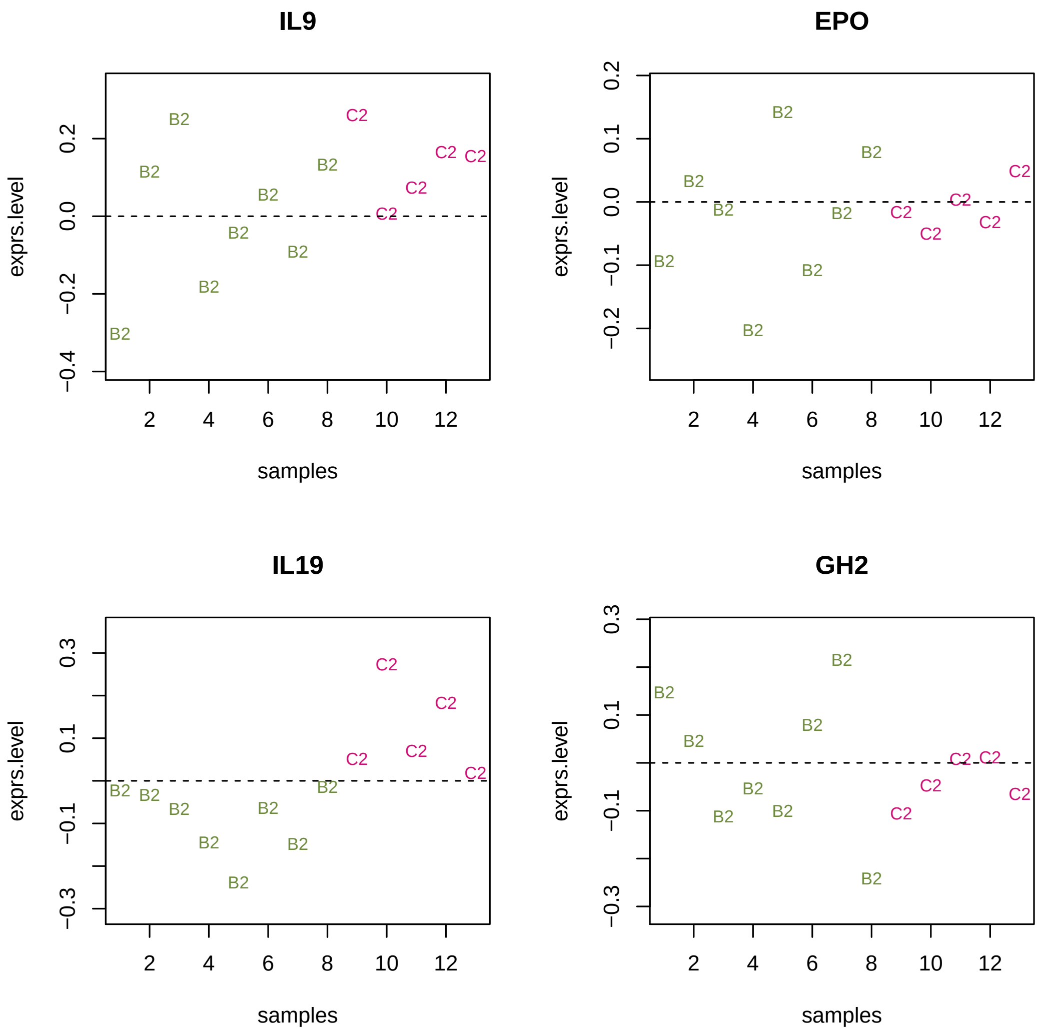

Plots of expression in all workers at time 2 of individual transcripts selected by Elastic Net at time 2

Analysis of Jak-STAT pathway genes with fold change

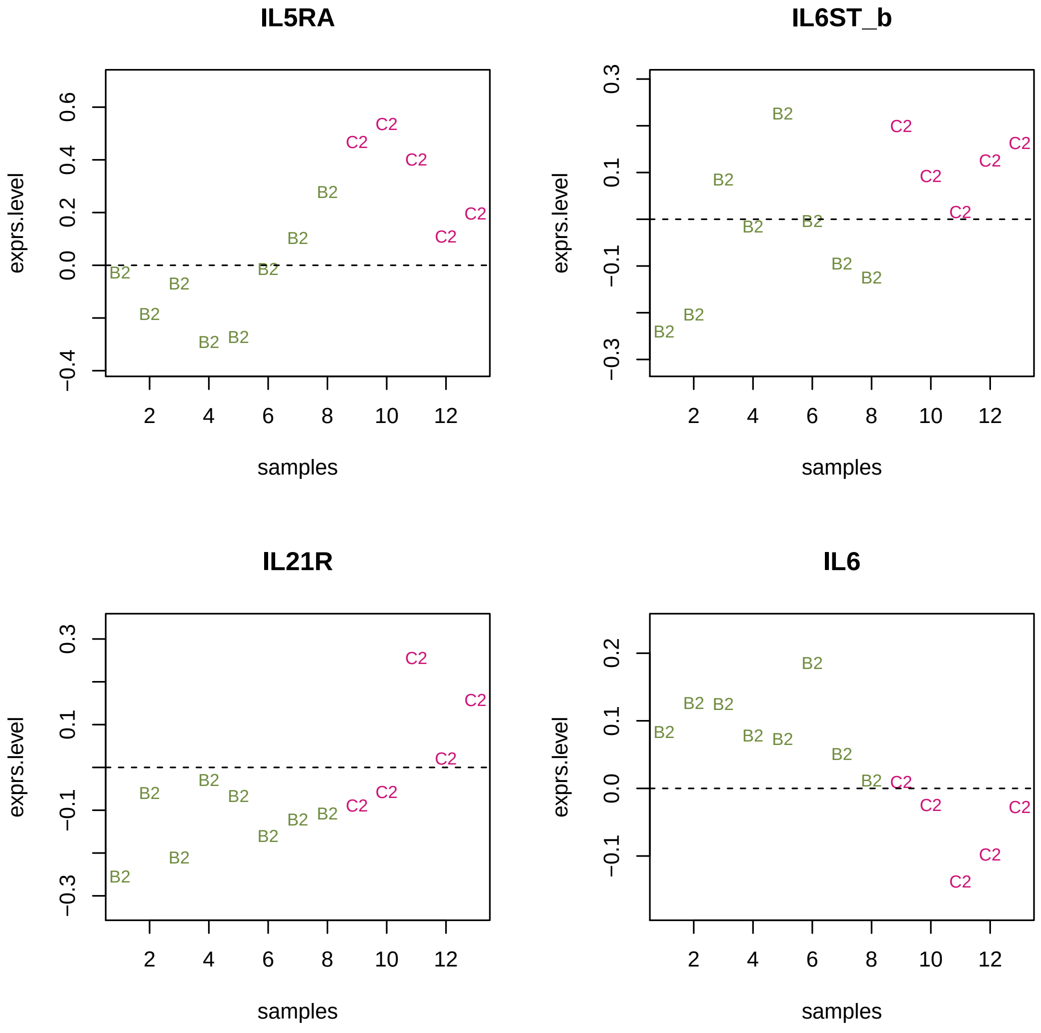

Plots of expression at time 2 in all workers of individual transcripts selected at time 2

Analysis of Jak-STAT pathway genes with fold change

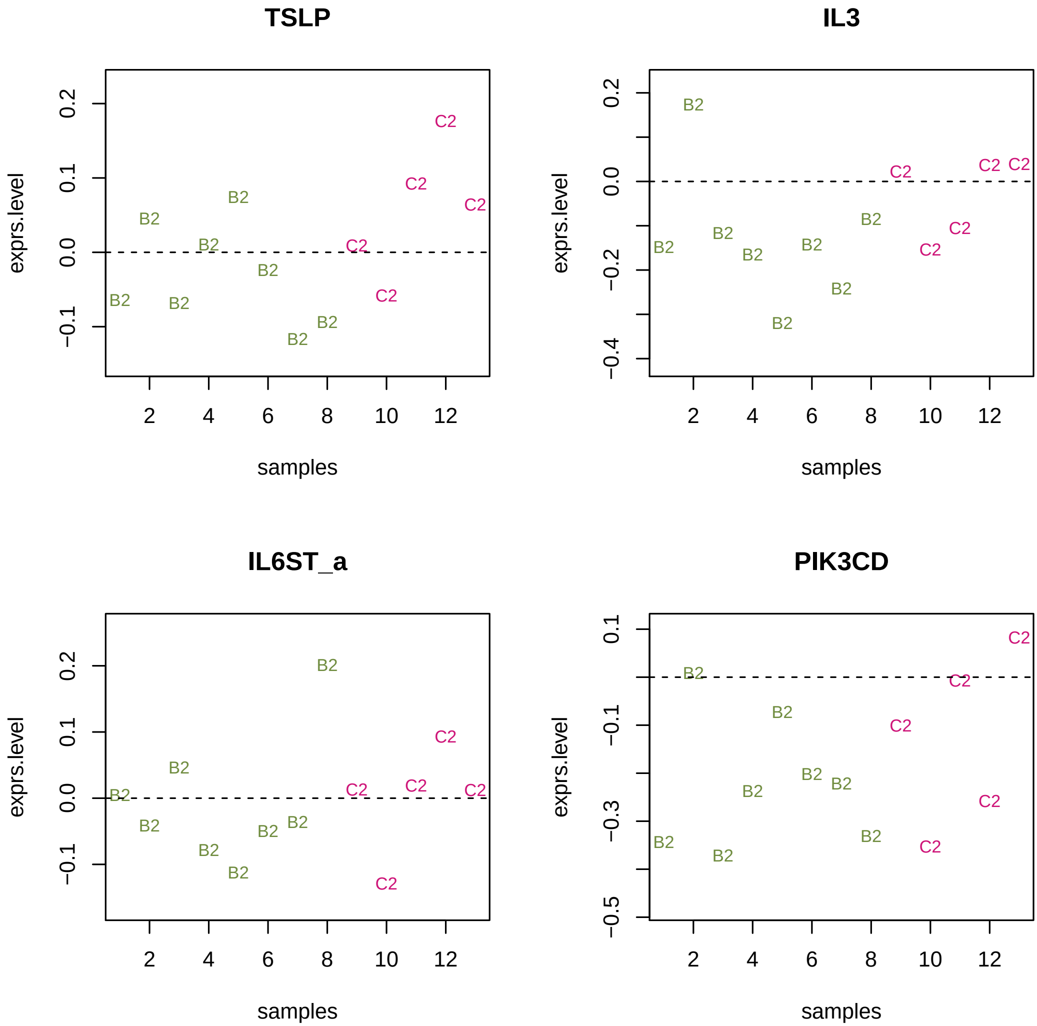

Plots of expression at time 2 in all workers of individual transcripts selected at time 2

Analysis of Jak-STAT pathway genes with fold change

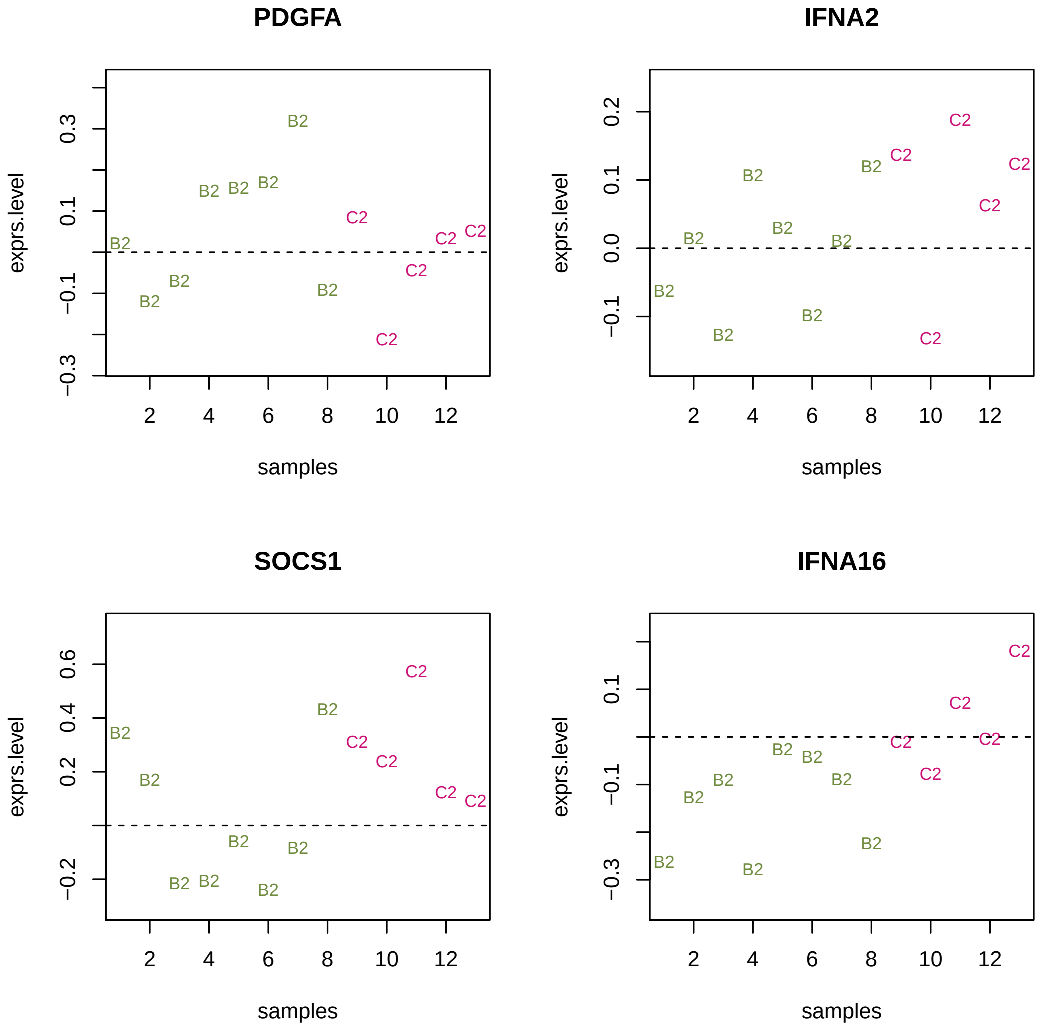

Plots of expression at time 2 in all workers of individual transcripts selected at time 2
