# Supplementary material for: Gene Expression Response in Peripheral Blood Cells of Petroleum Workers Exposed to Sub-Ppm Benzene Levels
Source: Int J Environ Res Public Health. 2018 Oct 27;15(11):2385. doi: 10.3390/ijerph15112385 (PMC6266895; doi:10.3390/ijerph15112385)
Supplement: Supplementary file 1 [file ijerph-15-02385-s001.zip › ijerph-344087-SI/Supplementary Information Nu/S7 Figure.pdf]

Gene plots of best pairwise markers in data with fold change

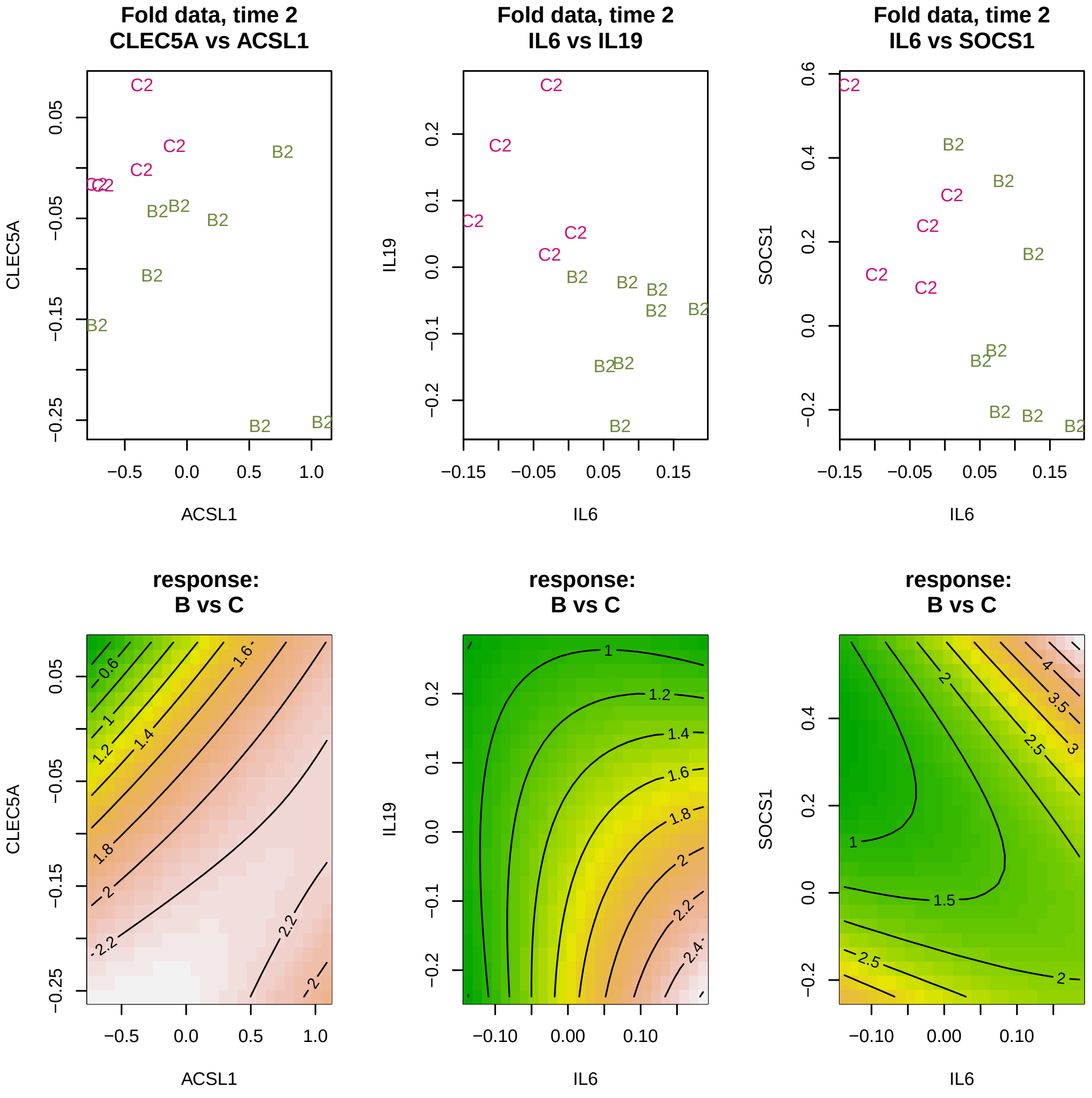

Plots of gene expression in all workers with fold change at time 2
Top: Pairwise plots Bottom: Response surface models

Gene plots of best pairwise markers in data with fold change

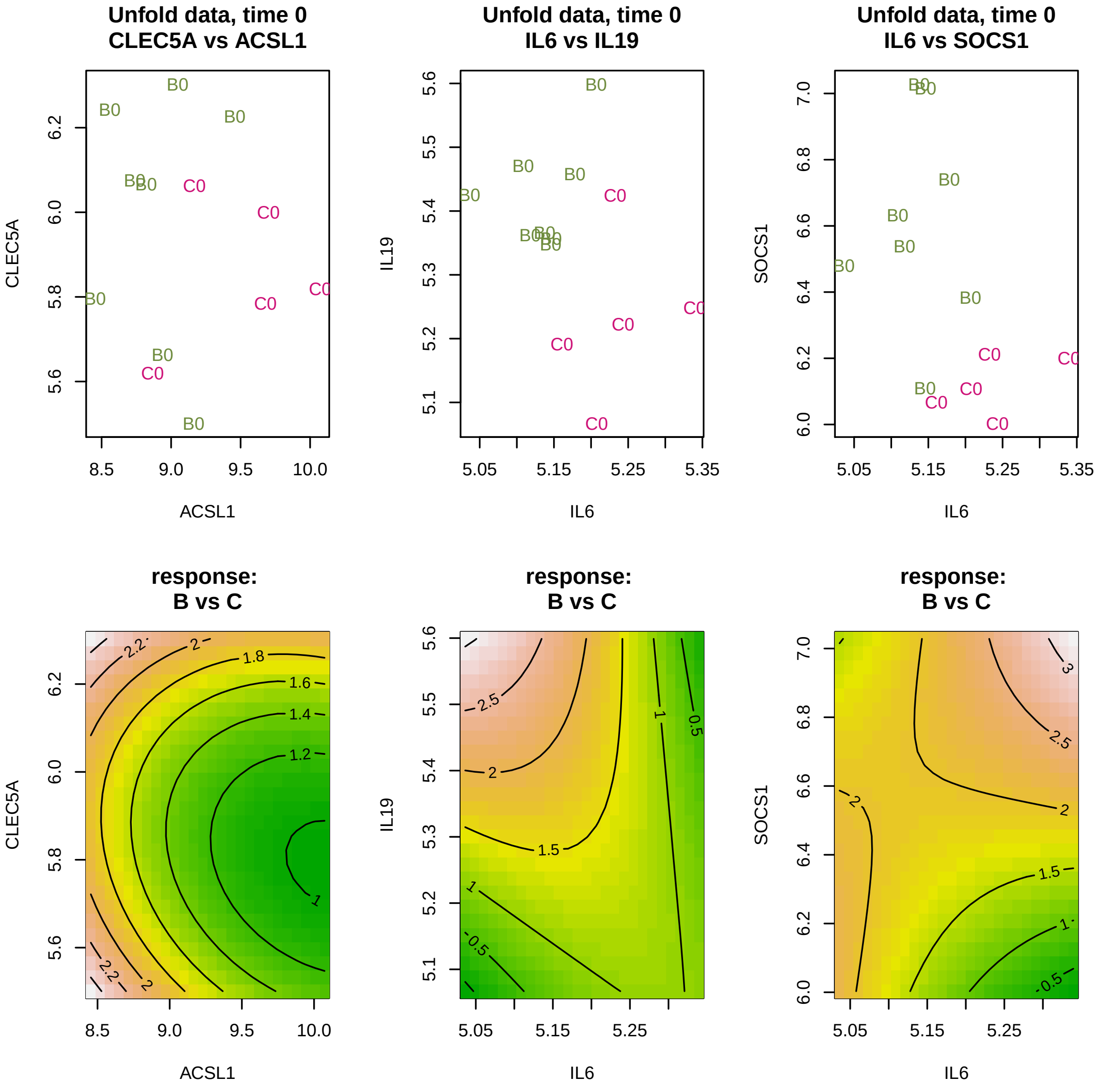

Plots of gene expression in all workers without fold change at time 0
Top: Pairwise plots Bottom: Response surface models
