# Supplementary material for: Gene Expression Response in Peripheral Blood Cells of Petroleum Workers Exposed to Sub-Ppm Benzene Levels
Source: Int J Environ Res Public Health. 2018 Oct 27;15(11):2385. doi: 10.3390/ijerph15112385 (PMC6266895; doi:10.3390/ijerph15112385)
Supplement: Supplementary file 1 [file ijerph-15-02385-s001.zip › ijerph-344087-SI/Supplementary Information Nu/S8 Figure.pdf]

Analysis of all 30 Schiffman marker genes without fold change

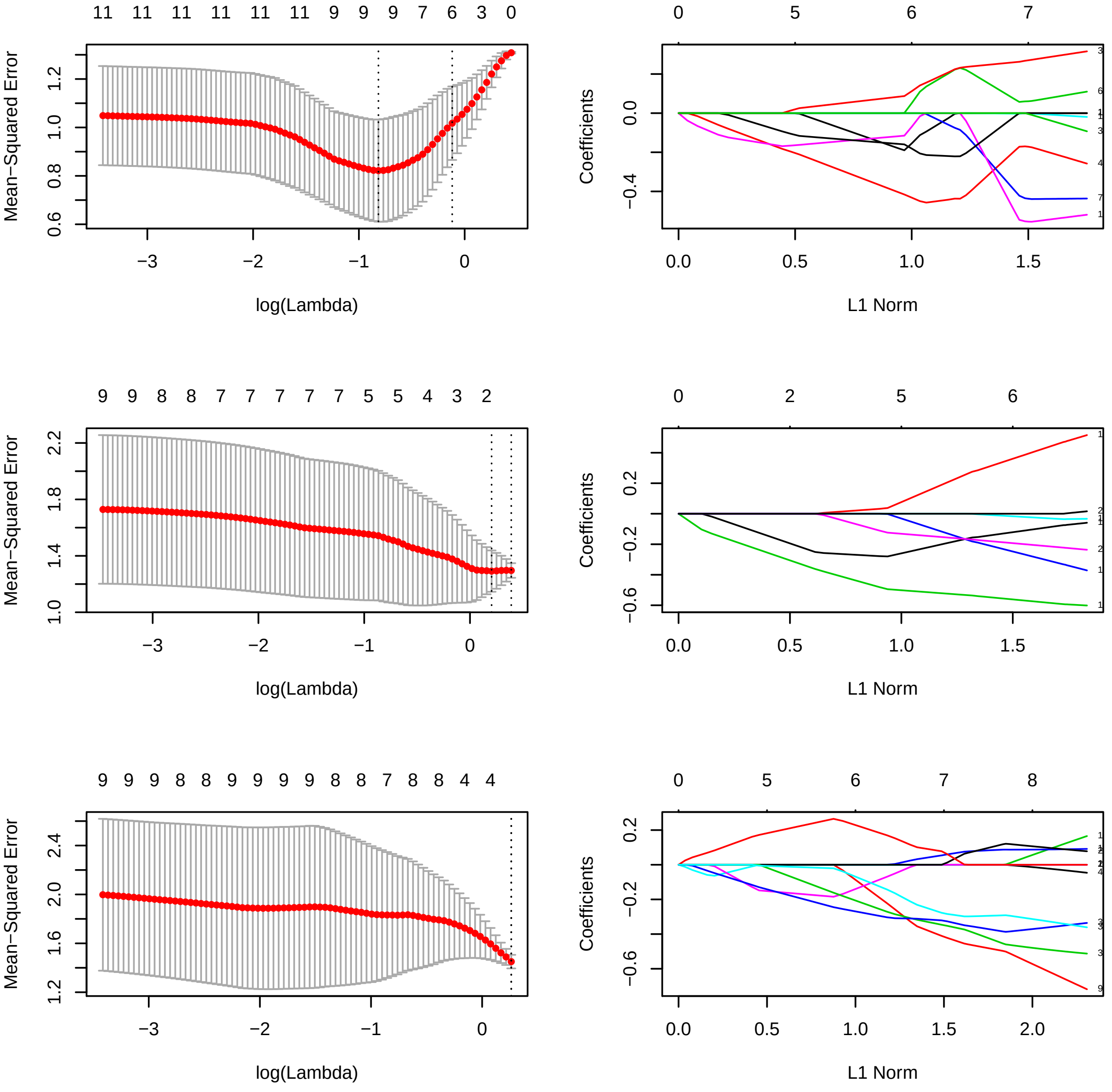

Elastic net results at time0 (top), 1 (middle) and 2 (bottom).
Left: Cross validation curves. Right: Coefficients for individual transcripts

Analysis of all 30 Schiffman marker genes without fold change

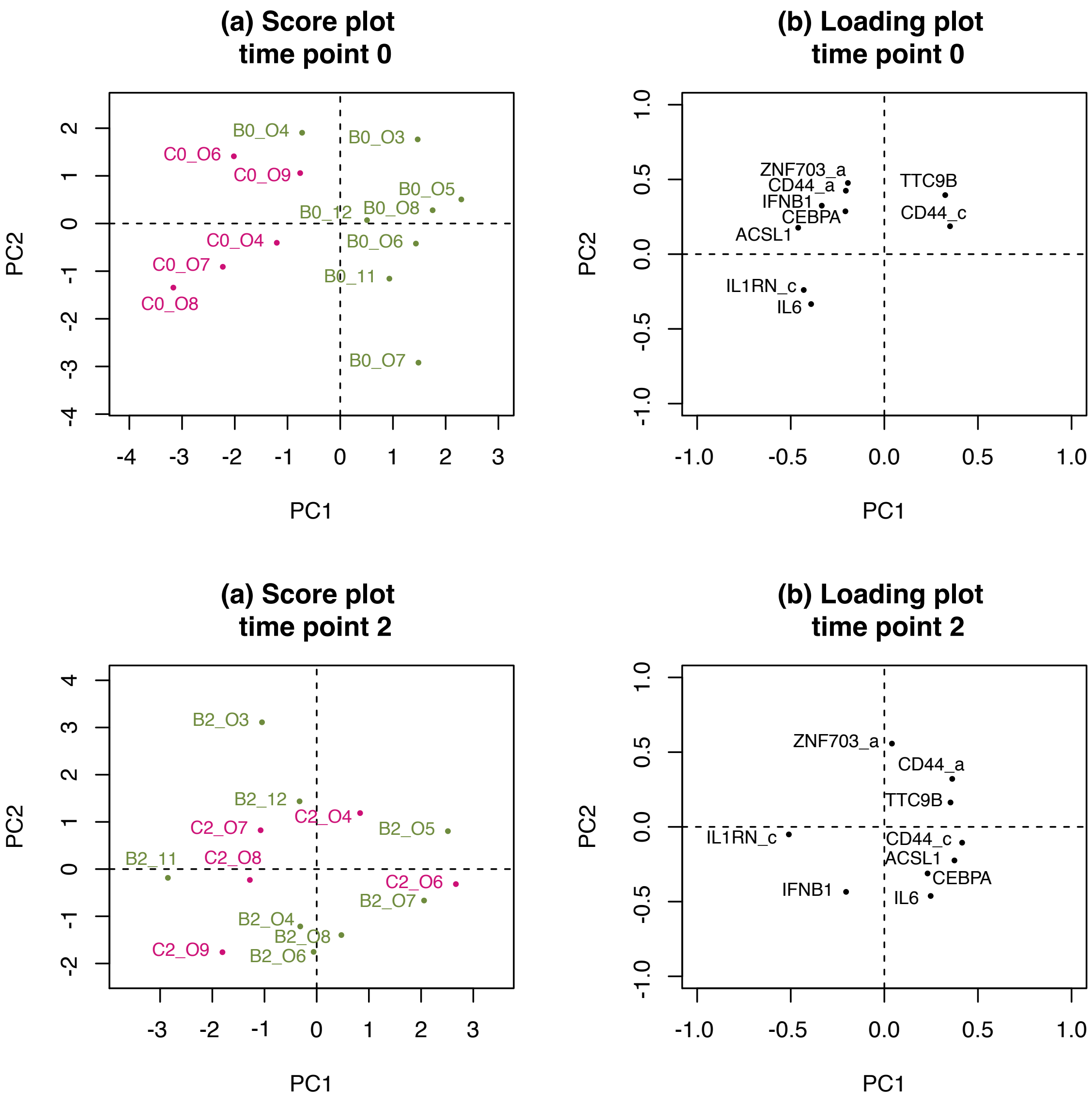

PCA of transcripts selected by Elastic Net at time 0
plotted at both time 0 (top) and time 2 (bottom)

Analysis of all 30 Schiffman marker genes without fold change

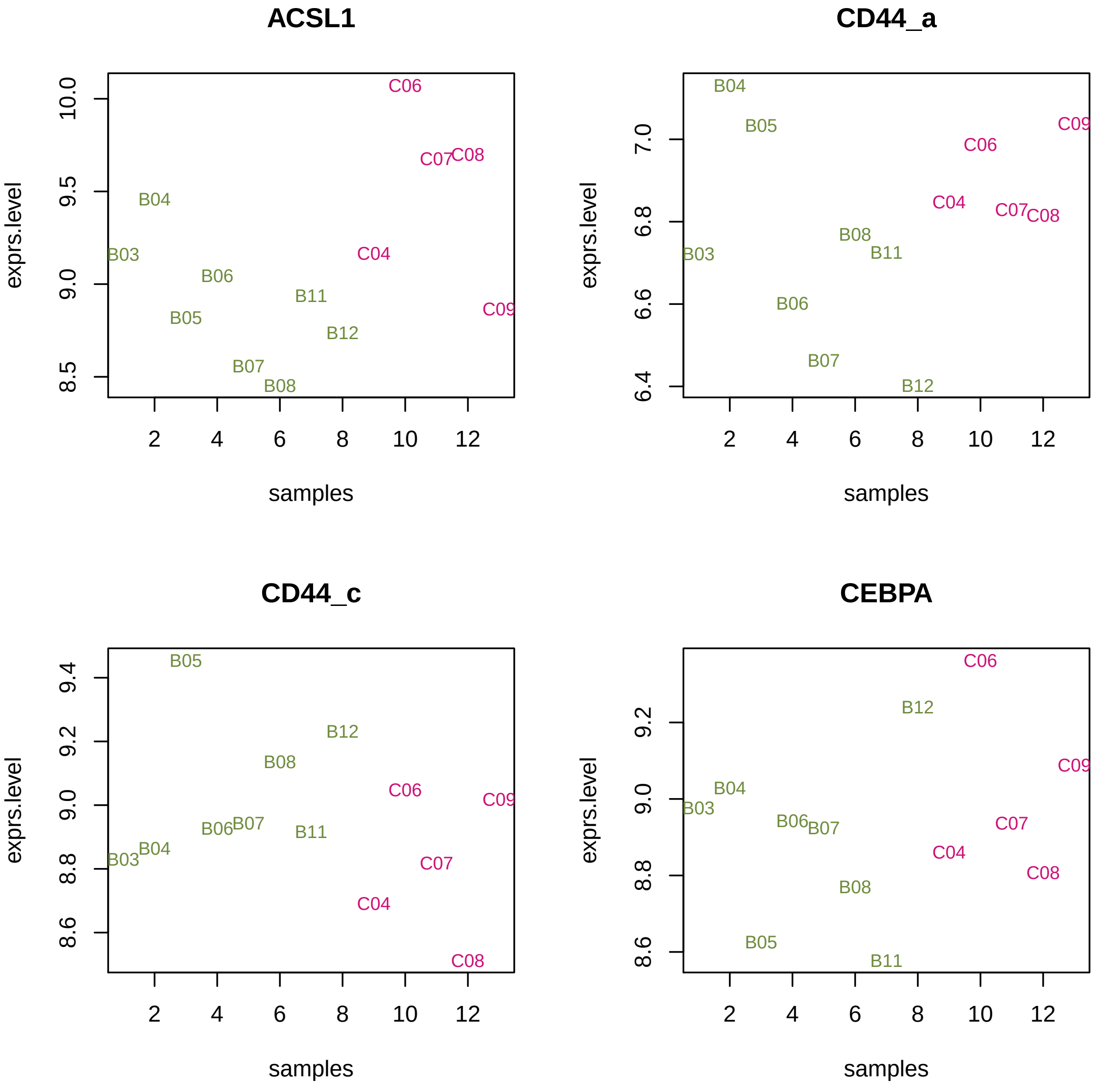

Plots of expression in all workers at time 0
of individual transcripts selected by Elastic Net at time 0

Analysis of all 30 Schiffman marker genes without fold change

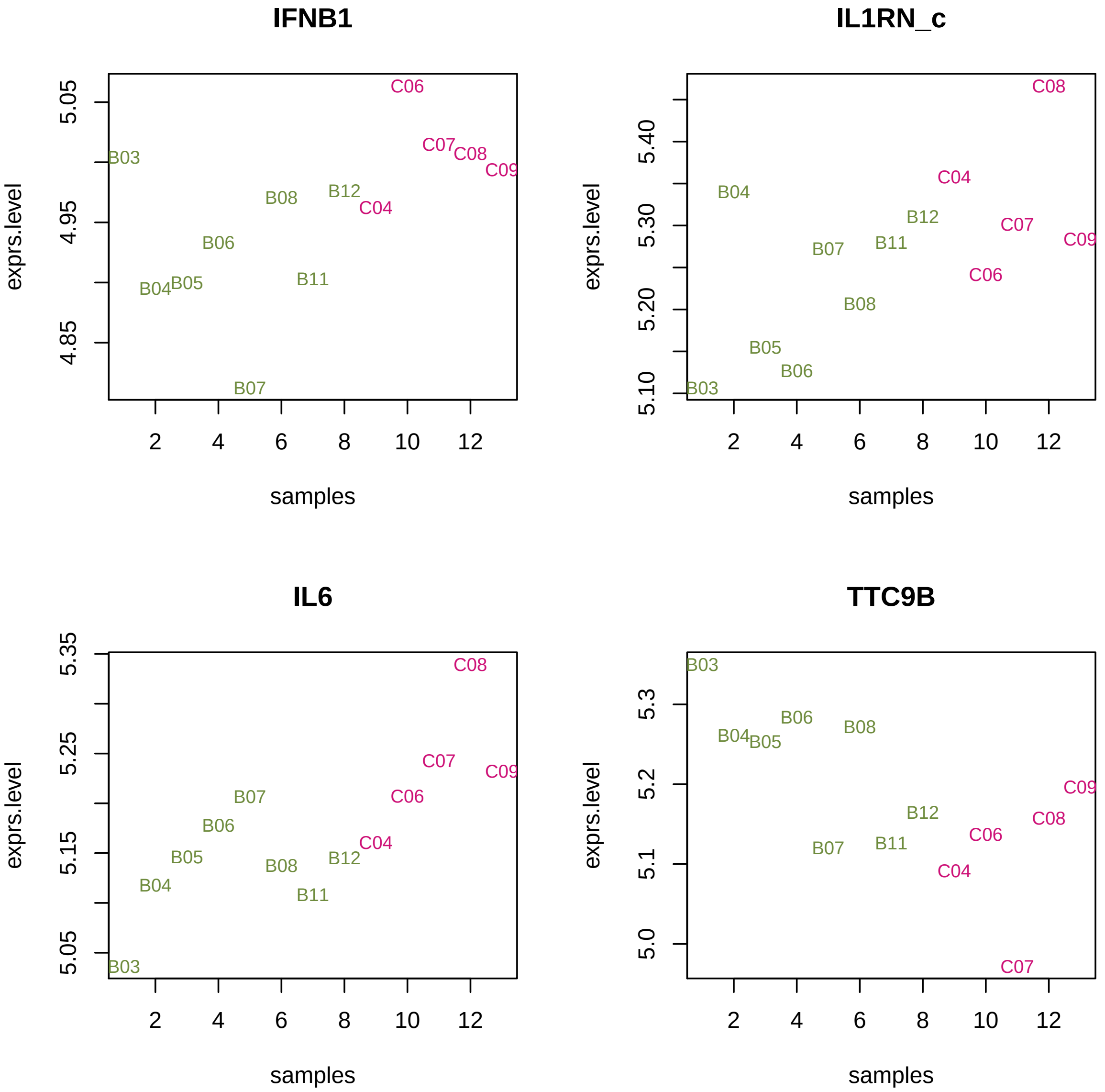

Plots of expression at time 0 in all workers
of individual transcripts selected by Elastic Net at time 0

Analysis of all 30 Schiffman marker genes without fold change

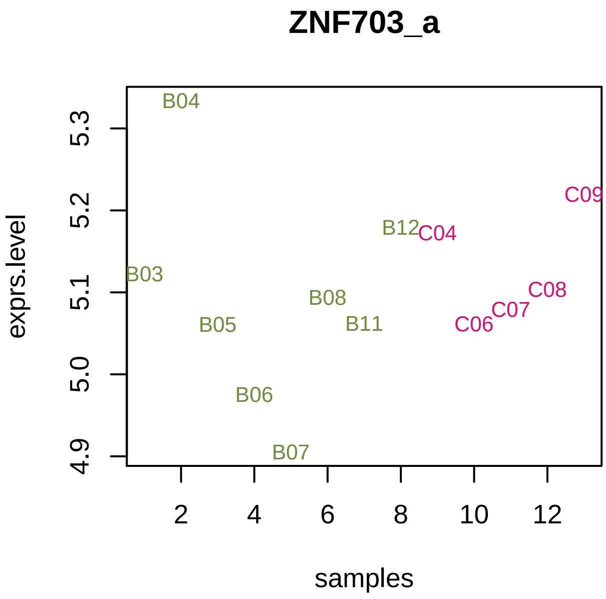

Plots of expression at time 0 in all workers of
individual transcripts selected by Elastic Net at time 0

Analysis of all 30 Schiffman marker genes without fold change

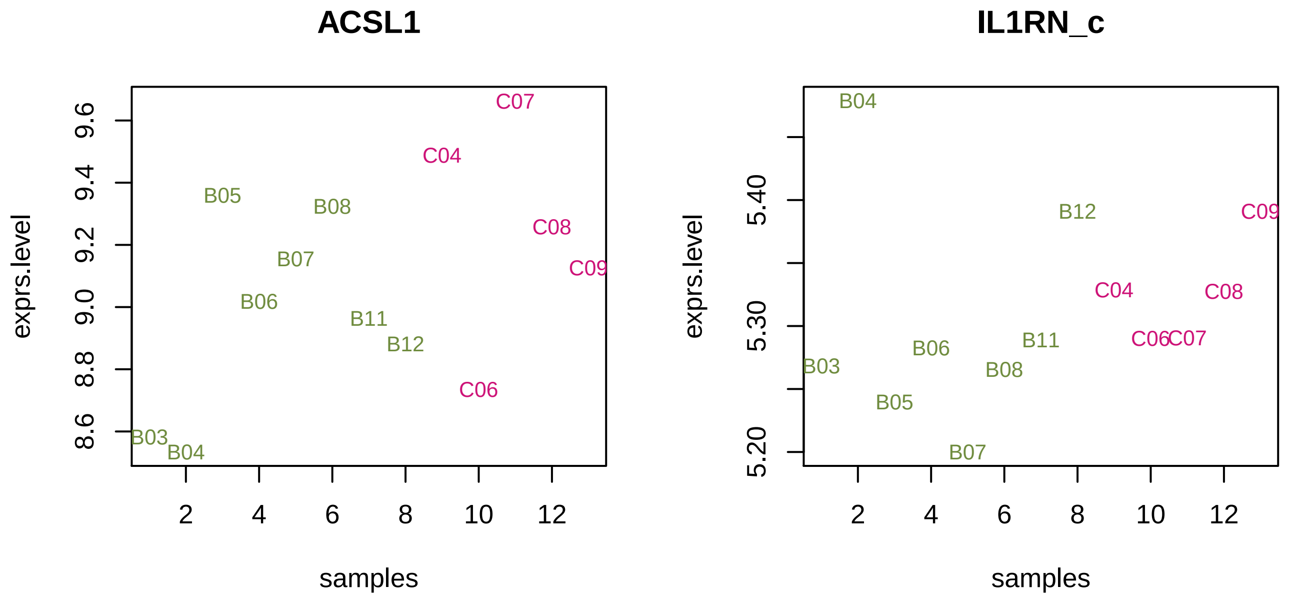

Plots of expression at time 1 in all workers of individual transcripts
selected by Elastic Net at time 1
