# Supplementary material for: Gene Expression Response in Peripheral Blood Cells of Petroleum Workers Exposed to Sub-Ppm Benzene Levels
Source: Int J Environ Res Public Health. 2018 Oct 27;15(11):2385. doi: 10.3390/ijerph15112385 (PMC6266895; doi:10.3390/ijerph15112385)
Supplement: Supplementary file 1 [file ijerph-15-02385-s001.zip › ijerph-344087-SI/Supplementary Information Nu/S9 Figure.pdf]

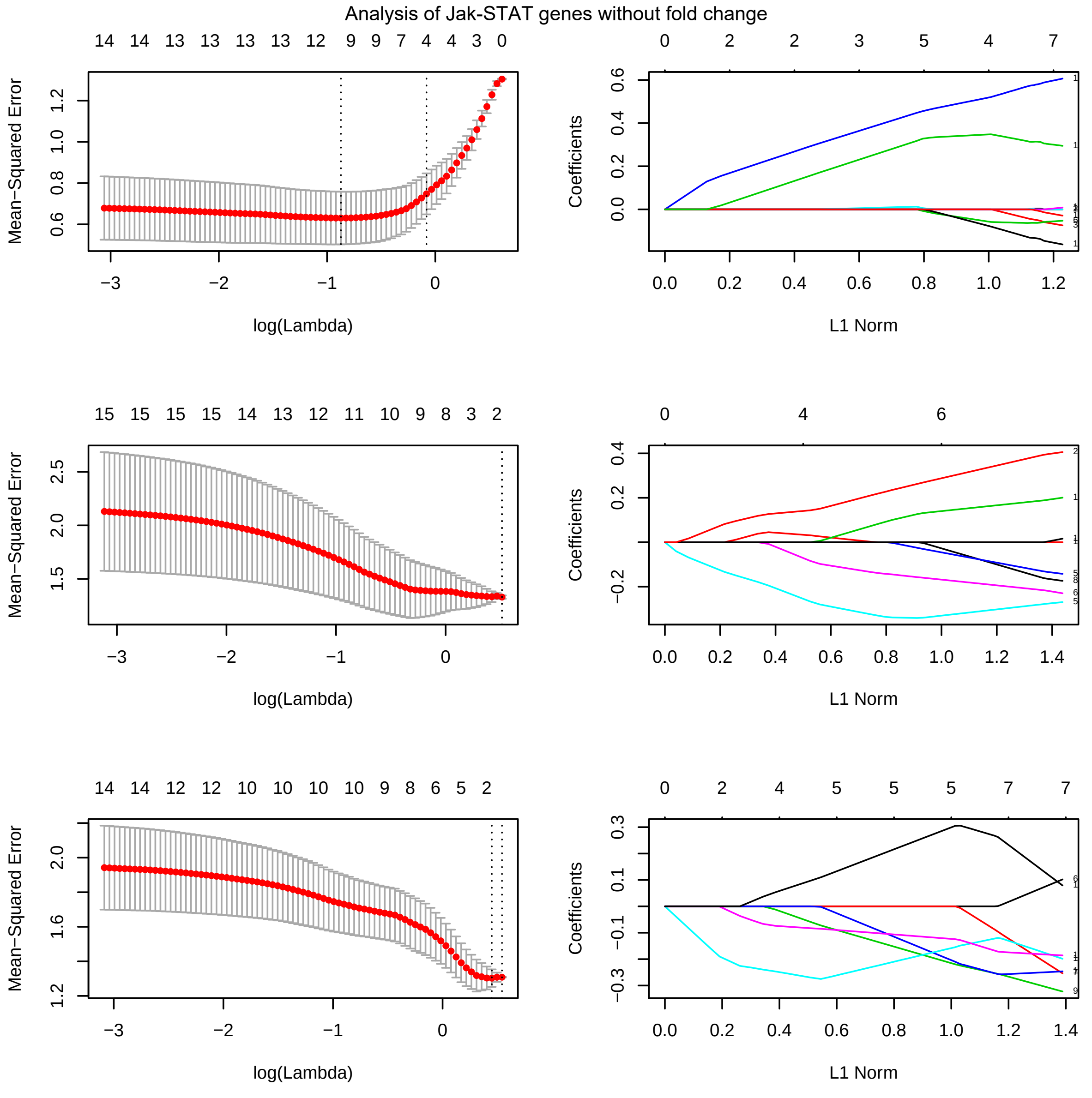


Elastic net results at time0 (top), 1 (middle) and 2 (bottom).
Left: Cross validation curves. Right: Coefficients for individual transcripts.

Analysis of Jak-STAT genes without fold change

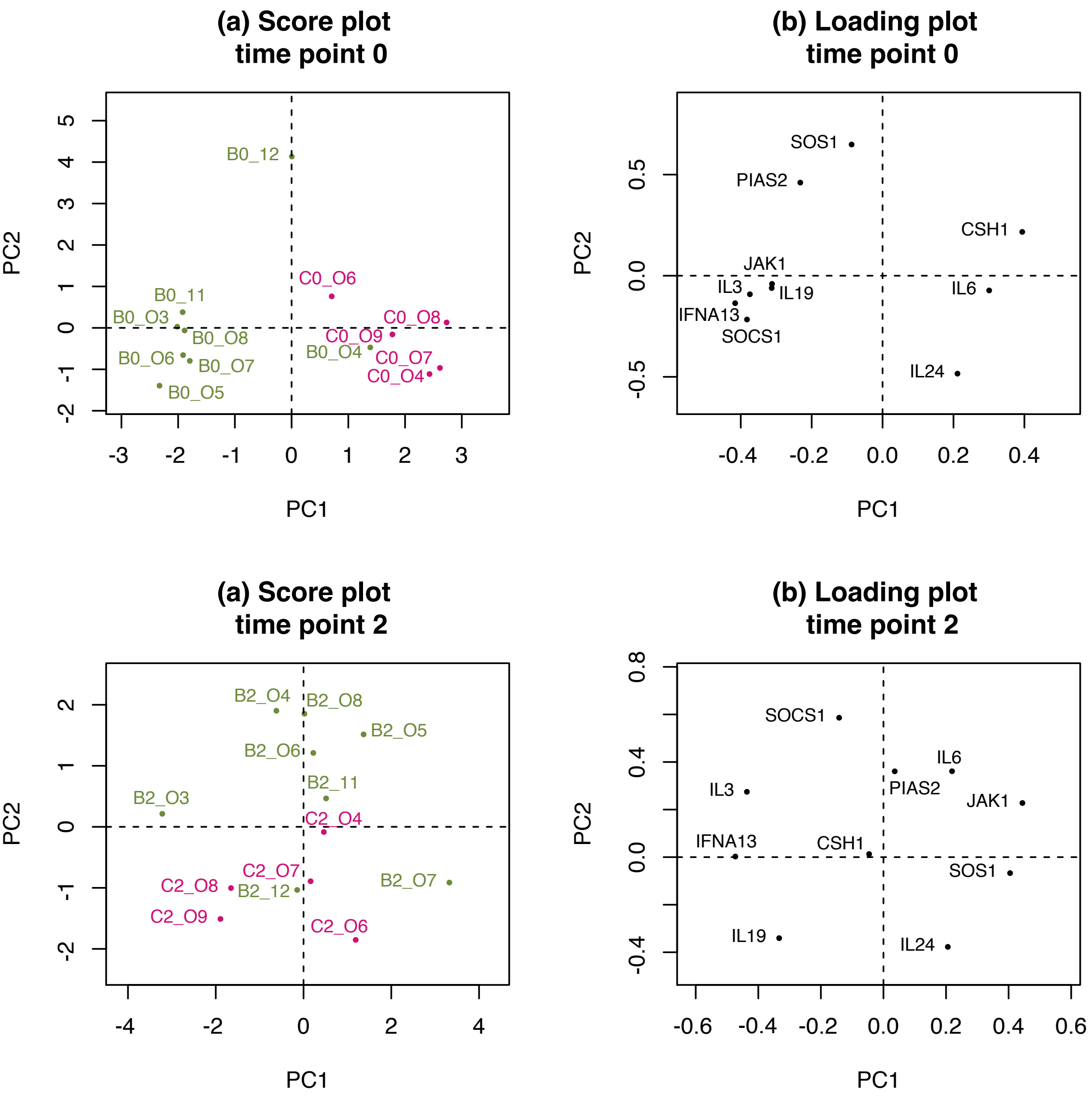


PCA of transcripts selected by Elastic Net at time 0 plotted at time 0 (top) and time 2 (bottom).

Analysis of Jak-STAT genes without fold change

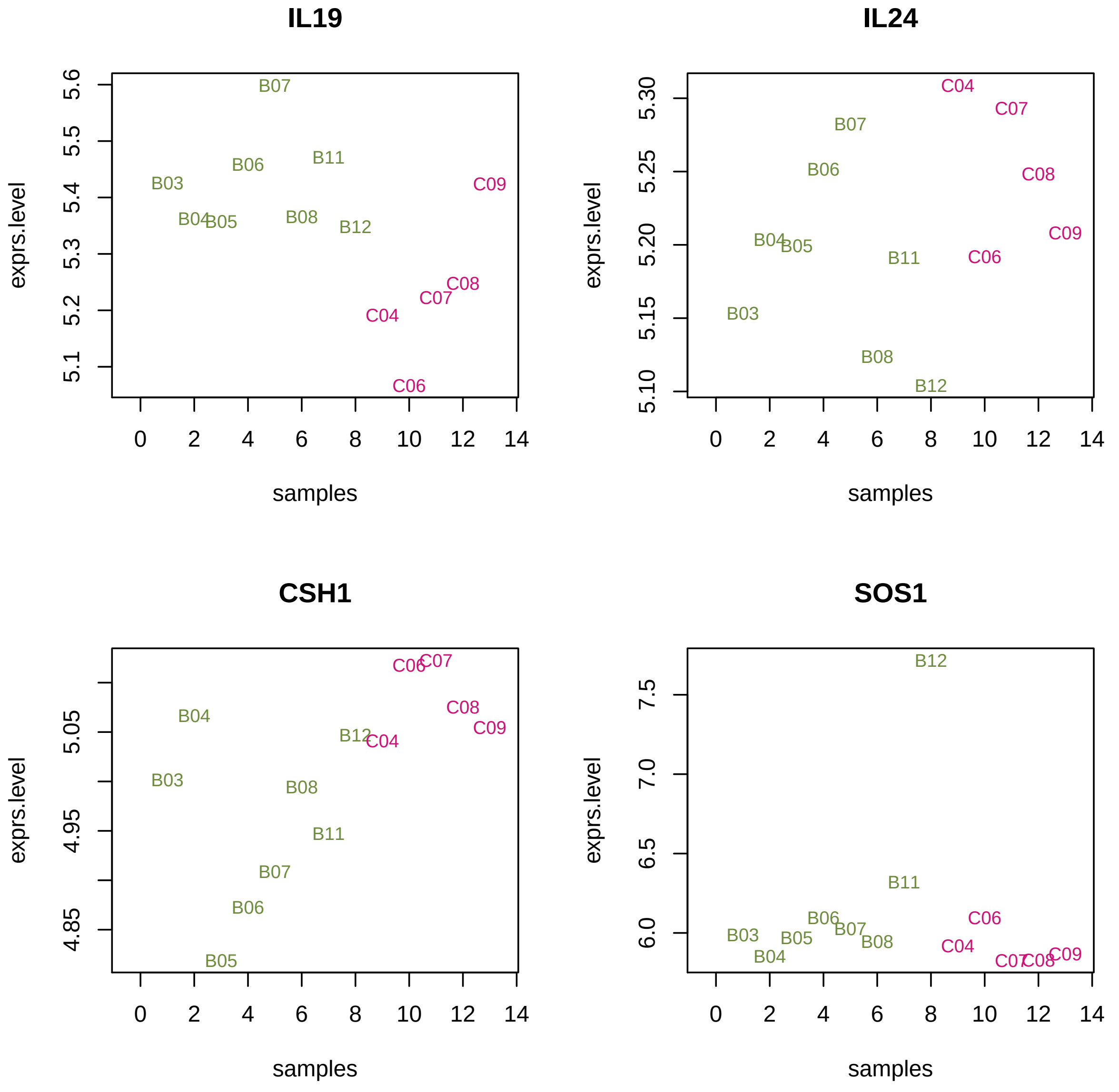


Plots of expression in all workers at time 0 for transcripts selected by Elastic Net at time 0

Analysis of Jak-STAT genes without fold change

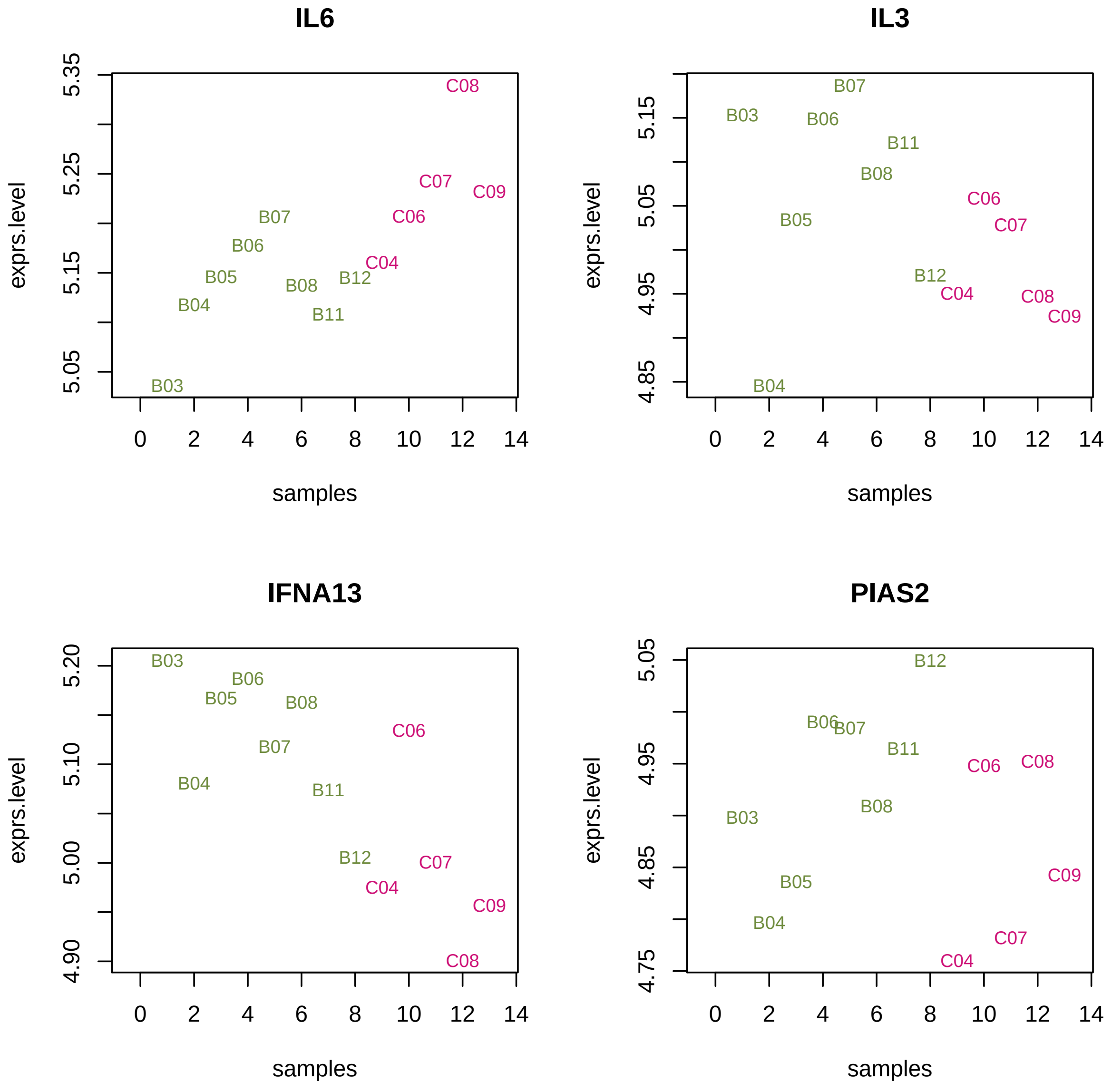


Plots of expression in all workers at time 0 for transcripts selected by Elastic Net at time 0

Analysis of Jak-STAT genes without fold change

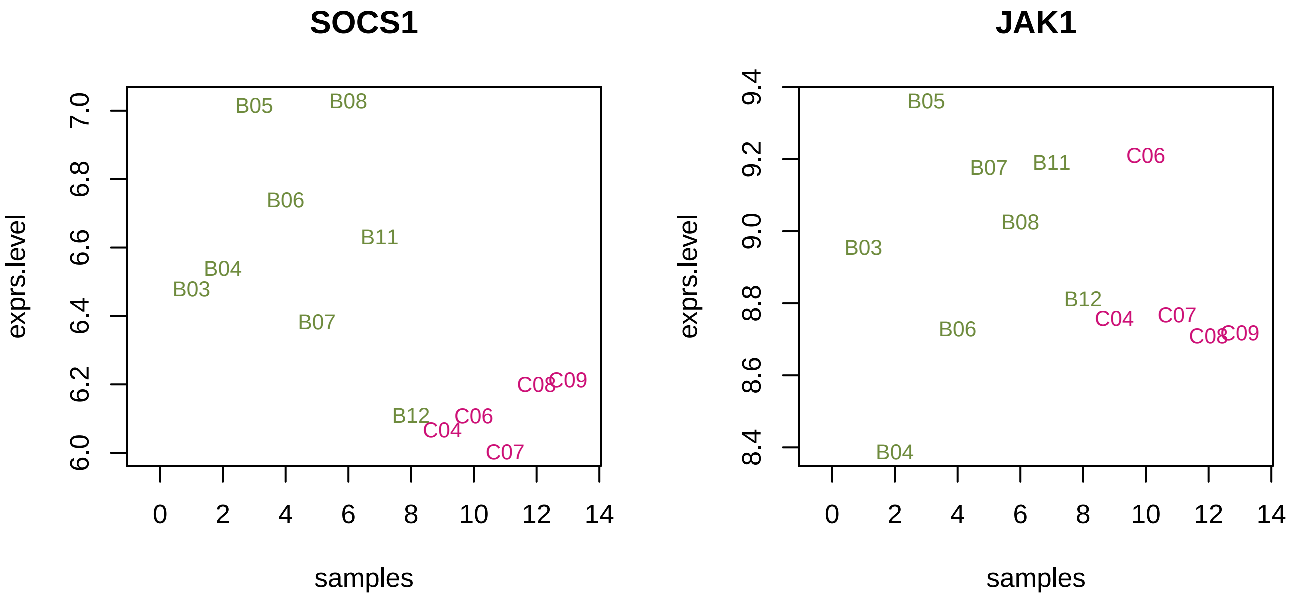


Plots of expression in all workers at time 0 for transcripts selected by Elastic Net at time 0

Analysis of Jak-STAT genes without fold change

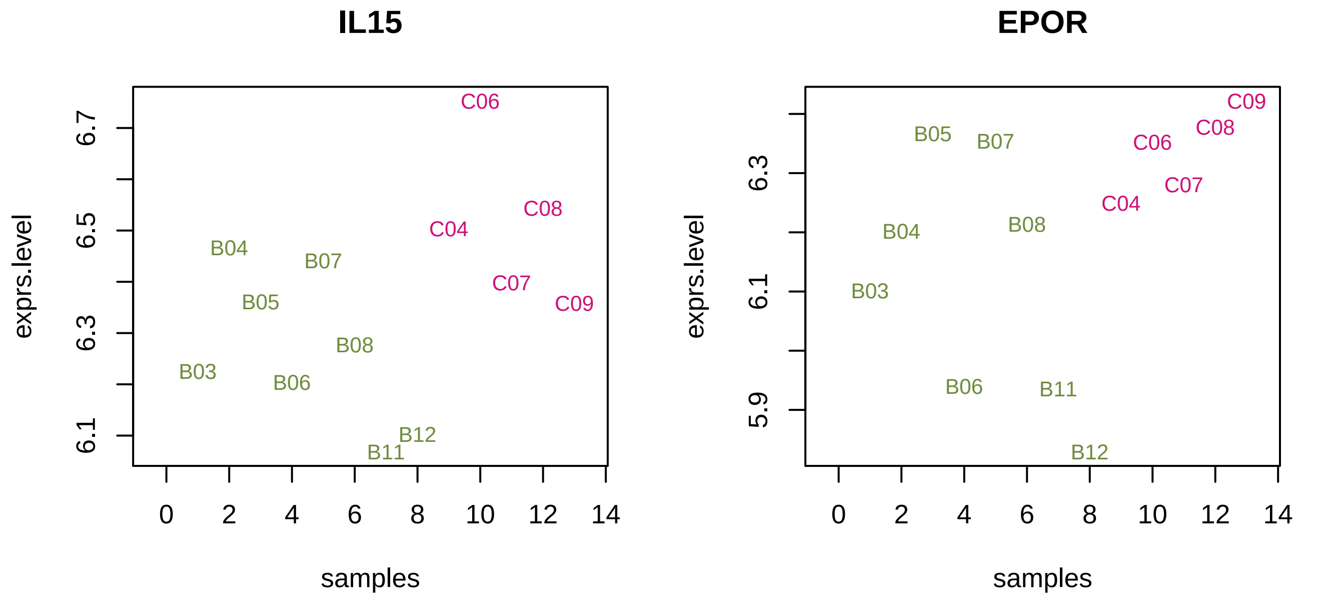


Plots of expression in all workers at time 2 for transcripts selected by Elastic Net at time 2
