# Supplementary material for: Gene Expression Response in Peripheral Blood Cells of Petroleum Workers Exposed to Sub-Ppm Benzene Levels
Source: Int J Environ Res Public Health. 2018 Oct 27;15(11):2385. doi: 10.3390/ijerph15112385 (PMC6266895; doi:10.3390/ijerph15112385)
Supplement: Supplementary file 1 [file ijerph-15-02385-s001.zip › ijerph-344087-SI/Suppl info corrected/S1a Table.doc]

# **SPØRRESKJEMA**

| PERSONALIA | | | | | | |
| --- | --- | --- | --- | --- | --- | --- |
| Etternavn, fornavn |  | | | | | |
| Kontaktadresse |  | | | | | |
| Telefon |  | | | | | |
| Kjønn |  | | | | | |
| Fødselsdato |  | | | | | |
| ARBEIDSHISTORIE | | | | | | |
| 2.1 Nåværende arbeidsgiver | |  | | | | |
| 2.2 Nåværende arbeidssted | |  | | | | |
| 2.3 Hvilkent yrke/tittel har du på dette arbeidsstedet? | |  | | | | |
| 2.4 Hvor lenge har du praktisert i dette yrket i ditt liv? | |  | | | | |
| Hvilke andre yrker har du hatt? Og hvor? | |  | | | |  |
|  | |  | | | |  |
|  | |  | | | |  |
|  | |  | | | |  |
| RØYKEVANER | | | | | | |
| 3.1 Hvor lenge er du vanligvis daglig i røykfylt rom? | | | | | Antall hele timer: 🞐🞐 | |
| 3.2 Røyker du selv? | | | | |  | |
| Sigaretter daglig?  Sigarer/sigarillos daglig?  Pipe daglig?  Aldri røykt daglig | | | | | Ja 🞐 Nei 🞐  Ja 🞐 Nei 🞐  Ja 🞐 Nei 🞐  (Sett kryss) 🞐 | |
| 3.3 Hvis du har røykt daglig tidligere, hvor lenge er det siden du sluttet? | | | | | Antall år: 🞐🞐 | |
| 3. 4 Hvis du røyker daglig nå eller har røykt tidligere: | | | | |  | |
| Hvor mange sigaretter røyker eller røykte du vanligvis daglig? | | | | | Antall sigaretter: 🞐🞐 | |
| Hvor gammel var du da du begynte å røyke daglig? | | | | | Alder i år: 🞐🞐 | |
| Hvor mange år til sammen har du røykt daglig? | | | | | Antall år: 🞐🞐 | |
| 4. KAFFE/TE/ALKOHOL | | | | | | |
| 4.1 Hvor mange kopper kaffe/te drikker du daglig? | | | | Antall kopper daglig:  Kaffi: 🞐🞐  Te: 🞐🞐 | | |
| 4.2 Nyter du alkohol i friperiodene dine? | | | | Ja 🞐 Nei 🞐 | | |
| 5. BRUK AV MEDISINER  Med medisiner mener vi alle slags medisiner, både:  med og uten resept, naturmedisin, vitaminer og mineraler  medisin som svelges, inhaleres eller injiseres, stikkpiller, salver, kremer eller dråper. | | | | | | |
| 5.1 Bruker du medisiner fast? Hvis ja, hvilke? | | |  | | | |
|  |  |  |  | | | |
|  |  |  |  | | | |
|  |  |  |  | | | |
| 5.2 Har du tatt medisiner de tre siste døgn før prøvetakingen  av blod og urin? Hvis ja, hvilke og hvor mye? | | |  | | | |
|  |  |  |  | | | |
|  |  |  |  | | | |
|  |  |  |  | | | |
|  |  |  |  | | | |
| Har du tidligere gjennomgått behandling for kreft (stråling, cellegift, m.v.). Hvis ja, hvilken behandling og når? | | |  | | | |
