# Supplementary material for: Gene Expression Response in Peripheral Blood Cells of Petroleum Workers Exposed to Sub-Ppm Benzene Levels
Source: Int J Environ Res Public Health. 2018 Oct 27;15(11):2385. doi: 10.3390/ijerph15112385 (PMC6266895; doi:10.3390/ijerph15112385)
Supplement: Supplementary file 1 [file ijerph-15-02385-s001.zip › ijerph-344087-SI/Suppl info corrected/S1b Table.doc]

# **QUESTIONNAIRE**

| PERSONALS | | | | | | |
| --- | --- | --- | --- | --- | --- | --- |
| Surname, firstname |  | | | | | |
| Adress |  | | | | | |
| Phone number |  | | | | | |
| Sex |  | | | | | |
| Date of birth |  | | | | | |
| OCCUPATIONAL HISTORY | | | | | | |
| 2.1 Present employer | |  | | | | |
| 2.2 Present workplace | |  | | | | |
| 2.3 What occupation/job title do you have on your present work engagement? | |  | | | | |
| 2.4 For how long have you been practicing this occupation? | |  | | | | |
| What other occupations have you had? And where? | |  | | | |  |
|  | |  | | | |  |
|  | |  | | | |  |
|  | |  | | | |  |
| RØYKEVANER | | | | | | |
| 3.1 For how long do you normally stay in a room with cigarett smoke during a day? | | | | | Number of hoursr: 🞐🞐 | |
| 3.2 Do you smoke? | | | | |  | |
| Cigarettes daily?  Cigars/cigarillos daily?  Pipe daily?  Have never smoked daily | | | | | Yes 🞐 No 🞐  Yes 🞐 No 🞐  Yes 🞐 No 🞐  (Tick) 🞐 | |
| 3.3 If you have been a daily smoker: how many years since you stopped? | | | | | Number of years: 🞐🞐 | |
| 3. 4 If you are a daily smoker or have been a daily smoker: | | | | |  | |
| How many cigarettes do you smoke or did you smoke daily? | | | | | Number of cigarettes: 🞐🞐 | |
| How old where you when you started to smoke daily? | | | | | Age in year: 🞐🞐 | |
| How many years in total have you smoked daily? | | | | | Number of years: 🞐🞐 | |
| 4. COFFEE/TEA/ALCOHOL | | | | | | |
| 4.1 How many cups of coffee/tea do you drink during a day? | | | | Number of cups:  Coffee: 🞐🞐  Tea: 🞐🞐 | | |
| 4.2 Are you drinking alcohol during your periods of leave? | | | | Yes 🞐 No 🞐 | | |
| 5. USE OF MEDICATIONS  With medications we mean all kinds of medications, including:   - *with and without a prescription, natural products, vitamins and minerals.* - *medications that is to be swallowed, inhaled or injected, suppositories, salves, creams and drops.* | | | | | | |
| 5.1 Are you on permanent medication? If yes; which medications do you use? | | |  | | | |
|  |  |  |  | | | |
|  |  |  |  | | | |
|  |  |  |  | | | |
| 5.2 Have you used any medication during the three days before sampling of blood and urine? If yes; what kind of medication and how much? | | |  | | | |
|  |  |  |  | | | |
|  |  |  |  | | | |
|  |  |  |  | | | |
| Have you been under treatment of cancer (radiation, chemotherapy, etc)?  If yes; what kind of treatment and when? | | |  | | | |
